# Alveolar Basal Cells Differentiate towards Secretory Epithelial- and Aberrant Basaloid-like Cells In Vitro

**DOI:** 10.3390/cells11111820

**Published:** 2022-06-02

**Authors:** Petra Khan, Julien Roux, Sabrina Blumer, Lars Knudsen, Danny Jonigk, Mark P. Kuehnel, Michael Tamm, Katrin E. Hostettler

**Affiliations:** 1 Department of Biomedicine and Clinics of Respiratory Medicine, University Hospital Basel, University of Basel, 4031 Basel, Switzerland; petra.khan@unibas.ch (P.K.); julien.roux@unibas.ch (J.R.); sabrina.blumer@unibas.ch (S.B.); michael.tamm@usb.ch (M.T.); 2 Swiss Institute of Bioinformatics, 4031 Basel, Switzerland; 3 Institute of Functional and Applied Anatomy, Hannover Medical School, 30625 Hannover, Germany; knudsen.lars@mh-hannover.de; 4 Biomedical Research in Endstage and Obstructive Lung Disease Hannover (BREATH), The German Center for Lung Research (DZL), 30625 Hannover, Germany; jonigk.danny@mh-hannover.de (D.J.); kuehnel.mark@mh-hannover.de (M.P.K.); 5 Institute of Pathology, Hannover Medical School, 30625 Hannover, Germany

**Keywords:** IPF, aberrant basaloid cells, basal cells, alveolar epithelial cells, differentiation, trans-differentiation, single cell RNA sequencing

## Abstract

In idiopathic pulmonary fibrosis (IPF), keratin (KRT)17+/KRT5+ basal and KRT17+/KRT5− aberrant basaloid cells are atypically present within the alveolar space. We previously described the fibrosis-enriched outgrowth of alveolar basal cells from peripheral fibrotic lung tissue. Using single cell RNA sequencing (scRNA-seq), we here characterize the transcriptome of these cultured alveolar basal cells under different culture conditions. Methods: Fibrotic peripheral lung tissue pieces were placed in DMEM growth medium. Outgrown cells were analysed by scRNA-seq, TaqMan-PCR or immunofluorescence (IF) either directly or after medium change to an epithelial cell specific medium (Cnt-PR-A). Results: A fraction of alveolar basal cells cultured in DMEM growth medium showed close transcriptomic similarities to IPF basal cells. However, although they expressed KRT5, the transcriptome of the majority of cells matched best to the transcriptome of recently described KRT17+/KRT5− aberrant basaloid cells, co-expressing the canonical basal cell marker KRT17 and mesenchymal cell marker (VIM, FN1). A smaller fraction of cells matched best to secretory epithelial cells. Two differentiation gradients from basal to aberrant basaloid-like cells and basal to secretory epithelial-like cells were apparent. Interestingly, these differentiation paths seemed reversed when the cell culture medium was changed to Cnt-PR-A. Conclusions: Our results suggest that cultured alveolar basal cells have the capacity to differentiate towards secretory epithelial-like cells and to aberrant basaloid-like cells. However, due to the persistent expression of KRT5, a complete differentiation towards aberrant basaloid cells did not seem to be achieved in our culture conditions. Importantly, differentiation seemed reversible by changing the cells microenvironment. Determining specific factors influencing these differentiation paths may help to define novel drug targets for IPF therapy.

## 1. Introduction

Idiopathic pulmonary fibrosis (IPF) is a rare, chronic, and irreversible interstitial lung disease (ILD) with progressive destruction of the lung parenchyma, resulting in loss of lung function [1]. Evidence suggests that repeated injuries to the alveolar epithelial cells play an important role in the development and progression of IPF [2]. In the healthy lung, the alveoli are lined with alveolar epithelial cells type 1 and 2 (AT1 and 2). AT2 cells maintain the alveolar epithelium by their ability to self-renew and they serve as progenitors for AT1 cells [3]. The conducting airway is lined with basal cells and different secretory and ciliated epithelial cells. Basal cells, expressing specific markers such as keratin (KRT)5, KRT17, KRT14, TP63 or integrin subunit beta (ITGB) 4, have the ability to differentiate into all other types of airway epithelial cells, and therefore serve as progenitors to regenerate the tracheobronchial epithelium after injury [4]. During normal lung homeostasis there is no overlap of epithelial cell types of the alveolar and the conducting regions. 

However, in severely injured regions of the peripheral IPF lung, a maladaptive repair program, involving a loss of AT2 cells accompanied with the accumulation of basal cells within the alveolar compartment, is apparent [5,6,7,8,9]. Similarly, severe lung injury in mice through H1N1 influenza infection or bleomycin resulted in an accumulation of basal-like cells in the alveolar region [10,11]. The function and origin of these atypical alveolar basal cells remains largely unknown. In mice, it was suggested that these cells contribute to alveolar epithelium regeneration by trans-differentiation into AT2 cells [12,13]. On the contrary, in IPF patients the presence of basal cells in bronchoalveolar lavage (BAL) fluid or in the alveolar region of lung tissue [8,14,15] was associated with increased mortality [8] and pathological bronchiolization and honeycomb formation [8,16]. 

In mice, alveolar basal cells originate from specific intrapulmonary p63+ progenitor cells that, upon injury, migrate into the alveolar space where they differentiate into basal cells [10,11,17]. In the human lung, where basal cells are present throughout the airways, distal airway basal cells may migrate into injured alveolar regions. Alternatively, alveolar basal cells may originate from resident AT2 cells [18]. Indeed, it was recently shown that AT2 cells have the ability to trans-differentiate into basal cells via SFTPC+/KRT17+ and SFTPC−/KRT17+/KRT5− alveolar-basal-intermediate (ABI1 and ABI2 respectively) cells in vitro. This process may also occur in the peripheral lung of IPF patients [5].

Besides alveolar basal cells, a rare aberrant basaloid cell population has recently been described in the lung of IPF patients. It is characterized by expression of the canonical basal cell marker KRT17, but not KRT5, along with mesenchymal cell markers, senescence markers, and IPF-associated molecules [6,7]. Interestingly, these aberrant basaloid cells show transcriptomic similarities to the above-mentioned ABI1 and ABI2 cells [5], and KRT8+ alveolar differentiation intermediate cells (ADI) [19]. This suggests that they may represent a transitional/intermediate cell type arising during AT2 to basal cell trans-differentiation and during AT2 to AT1 differentiation.

We previously reported the fibrosis-enriched outgrowth of a distinct cell population from the alveolar parenchyma of ILD patients in vitro [20], showing expression of the canonical basal cell markers KRT5 and KRT17 [9]. In this study, using scRNA-seq we investigated the transcriptomic landscape of our cultured alveolar basal cells under different culture conditions, and compared them to the previously characterized transcriptomes of basal, aberrant basaloid or transitional cells.

## 2. Materials and Methods

### 2.1. Cell Culture

Alveolar basal cells were cultured from peripheral fibrotic lung tissue in DMEM growth medium (DMEM supplemented with 10% FCS, 25 mM HEPES, 1× (*v*/*v*) sodium pyruvate, 1× (*v*/*v*) MEM vitamin-mix, and 1× (*v*/*v*) antibiotic-antimycotic) as previously described [9]. Briefly, peripheral fibrotic lung tissue was cut into small pieces and placed into 12-well culture dishes (one piece/well) containing DMEM growth medium. After 5 days the tissue pieces were removed, and cells harvested for downstream analysis. Cells derived from individual tissue pieces of the same patient were pooled in order to get sufficient cell numbers. In some experiments, DMEM growth medium was changed after five days to the commercially available, fully defined low calcium epithelial cell specific growth medium Cnt-prime airway (PR-A) and the cells grown in Cnt-PR-A for another five days prior to downstream analysis. IPF was diagnosed based on ATS/ERS guidelines [1,21]. The local ethical committee of the University Hospital, Basel, Switzerland (EKBB05/06) and of the Hannover Medical School, Germany (2699–2015) approved the culture of human primary lung cells. All materials used in this study are listed in Supplement Table S1.

### 2.2. Single Cell RNA Sequencing

Single-cell suspensions were generated from cells grown from cultured peripheral lung tissue biopsies from three IPF patients (IPF patient 1–3) in DMEM growth medium for 5 days, controlled for viability, and loaded on separate wells of a 10× Genomics Chromium Single Cell Controller in two batches (IPF patient 1 on 8 April 2019, and patients 2 and 3 on 27 May 2019) for an expected recovery of 10,000 cells.

A last single-cell suspension from cells originating from IPF patient 2 and transferred to a Cnt-PR-A growth medium after 5 days of culture in DMEM growth medium was loaded on a single well of the 10× Genomics Chromium Single Cell Controller on 27 May 2019, for an expected recovery of 10,000 cells.

Single cell capture and cDNA and library preparation were performed at the Genomics Facility Basel of the ETH Zurich, Basel with a Single Cell 3′ v3 Reagent Kit (10× Genomics) according to the manufacturer’s instructions. Sequencing was performed on one flow-cell of the Illumina NexSeq 500 platform (with 56nt-long R2 reads).

Read quality was assessed with the FastQC tool (version 0.11.5, Simon Andrews, Cambridge, UK). Sequencing files were processed with Kallisto (version 0.46.0) and BUStools (version 0.39.2) to perform sample and cell demultiplexing, and pseudo-alignment of reads to human transcripts (Ensembl release 96) and their intronic regions (extended by 50nt on each side). The commands “bustools correct”, “bustools capture” and “bustools count” were used to generate gene-level spliced and unspliced UMI counts table [22,23]. For the main analysis, the spliced UMI counts table was used.

Processing of the UMI counts matrix was performed using the Bioconductor packages DropletUtils (version 1.14.2) [24,25], scran (version 1.22.1) [26,27] and scater (version 1.22.0) [28], following mostly the steps illustrated in the OSCA book (http://bioconductor.org/books/release/OSCA/; last accessed on 20 May 2022) [27,29]. 

Based on the observed distributions, cells with 0% or more than 25% of UMI counts attributed to the mitochondrial genes [30], with less than 631 UMI counts, or with less than 502 detected genes were excluded. The presence of doublet cells was investigated with the scDblFinder package (version 1.8.0), and suspicious cells were filtered out (score > 0.95).

After quality filtering, the resulting DMEM growth medium dataset consisted of 8625 cells, including 7196 derived from patient 1, 887 cells from patient 2, and 542 cells from patient 3.

Cell cycle phase was inferred using the markers provided in Seurat (version 4.1.0) and the CellCycleScoring function. A proliferative signature was taken from Travaglini et al. [31]. This is calculated as the average expression of 11 genes (PBK, BIRC5, MKI67, UBE2C, TOP2A, TK1, AURKB, CDKN3, CENPF, CDK1, ZWINT) trimmed to the highest and lowest 0.1% scores for better plotting dynamics. A cutoff of 0.2 was used to call cells proliferating or not.

Systematic differences between samples were removed using the fastMNN function (d = 50, k = 20) of the batchelor package (version 1.10.0) [32], run on the combined hypervariable genes across samples (954 genes in total). Two-dimensional *t*-distributed stochastic neighbor embedding (tSNE) used for visualization of cells was calculated using the 500 most variable genes and the batch-corrected matrix of low-dimensional coordinates for each cell (perplexity of 30). Clustering of cells was performed on the batch-corrected dataset with hierarchical clustering on the Euclidean distances between cells (with Ward’s criterion to minimize the total variance within each cluster; package cluster version 2.1.0). The number of clusters used for following analyses was identified by applying a dynamic tree cut (package dynamicTreeCut, version 1.63-1; deepSplit = 0). The scran function findMarkers was used to find markers up-regulated in any of the clusters. The top 10 markers for each clusters were extracted and pooled to form a list of 89 unique genes. The package SingleR (version 1.8.1) was used for unbiased cell-type annotation of the cells [33] using as reference the public scRNA-seq datasets described above. Only high-quality assignments (pruned scores) from SingleR were used. For co-expression plots of KRT5 or KRT17 with other cell-type specific markers, we used a cutoff of 0.5 on the normalized log-counts to account for potential background noise when determining the present/absent status.

Differentiation states across cells were inferred using the R package CytoTRACE (version 0.33.1, available at https://cytotrace.stanford.edu/ last accessed on 20 May 2022) [34]. RNA velocity calculations were performed based on the spliced and unspliced UMI count tables produced by Kallisto and BUStools for the cells of patient 1 only (to avoid misleading distortions induced by the batch integration methods). The package velociraptor (version 1.4.0) was used as a wrapper to the scvelo package (version 0.2.2) using the top hypervariable genes (filtered to remove genes high in ambient RNA pool, mitochondrial genes and ribosomal genes; 403 genes in total). The likelihood-based dynamical velocities were calculated using default parameters.

Processing of the Cnt-PR-A growth medium sample was performed similarly as described above, apart from no batch integration step. After quality filtering, the resulting dataset consisted of 5280 cells.

### 2.3. Reanalysis of Public scRNA-Seq Datasets

The dataset of human pulmonary fibrosis and control patients [35] was downloaded from GEO (accession GSE122960; HDF5 processed data files produced by CellRanger v2.0). Processing of the UMI counts matrix was performed using the Bioconductor packages DropletUtils (version 1.5.4) [24,25], scran (version 1.12) [26,36] and scater (version 1.12) [28], following mostly the steps illustrated in the OSCA Bioconductor book (https://bioconductor.org/books/release/OSCA/; last accessed on 20 May 2022) [27]. The resulting filtered and normalized expression matrix included 14,983 genes and 108,509 cells from 17 patients. Clustering of cells was done on normalized log-count values using a shared nearest-neighbours graph approach (scran function buildSNNGraph), resulting in 93 clusters. Annotation of cell type and identification of clusters of cell doublets was done based on the expression of known marker genes and following the annotation of the original study [35] (Supplement Figure S1). Clusters of cell doublets and clusters of immune cells were filtered out for further analyses.

The IPF Cell Atlas dataset from Adams et al. [6] was downloaded from GEO (accession GSE136831; R “.rds” files including exonic UMI count matrices for each patient, and the “GSE136831_AllCells.Samples.CellType.MetadataTable.txt.gz” file including the annotation for cells). Only non-immune cells from control and IPF patients were retained. We additionally excluded cells from patients with less than 50 cells left. The final dataset was composed of 30,617 cells, 7898 from control patients, and 22,719 from IPF patients.

The Habermann et al. dataset [7] was downloaded from GEO (accession GSE135893; Full Seurat object downloaded as “.rds” file). Only non-immune cells from control and IPF patients were retained. The final dataset was composed of 44,137 cells, 12,151 from control patients, and 31,986 from IPF patients.

The atlas of cell types in the human lung from Travaglini et al. [31] was downloaded from https://www.synapse.org/#!Synapse:syn21041850/files/ (processed UMI count matrix and annotation of cells; last accessed on 20 May 2022). For simplicity the different subsets of immune cells were grouped, as well as the fibroblast/myofibroblast/smooth muscle subsets. We also ignored the site of sampling (proximal, medial or distal lung).

The dataset from Kathiriya et al. [5] of cultured AT2 epithelial cells undergoing transdifferentiation in vitro towards basal cells organoids was downloaded from GEO (accessions GSE150068 and GSE150247). The outputs from CellRanger v2 (day 0, sample “NL_2”, and day 14, sample “Day14_Organoid”) or v3 (day 7, sample “Day7_Organoid”, and day 21, sample “Day21_Organoid”) were used to gather the UMI count matrix and the clustering and annotation metadata for the cells was obtained from the authors upon E-mail request.

The mouse dataset from Strunz et al. [19] was obtained from GEO (accession GSE141259). The processed data files for the epithelial cells dataset (“HighResolution” dataset) were used. The clustering and annotation metadata for the cells was obtained from the authors upon E-mail request. For annotation of our cells with SingleR, the correspondence from mouse to human Ensembl IDs was made by retrieving the 1-to-1 orthologs between human and mouse from Herrero et al. 2016 [37].

### 2.4. TaqMan RT-PCR, Immunofluorescence & Electron Microscopy

TaqMan^®^ PCR and immunofluorescence (IF) stainings were performed as previously described [20,38]. Details for primer and antibodies are listed in Supplement Table S1. For electron microscopy (EM) imaging, cells were fixed in 4% paraformaldehyde/0.1% glutaraldehyde/0.2 M HEPES and embedded in epoxy resin (Epon^®^) and subjected to EM imaging as previously described [39]. Semi-thin sections were cut and stained with toluidine blue for light microscopic investigation. Afterwards, ultrathin sections were used for transmission electron microscopy (Morgagni, FEI, Eindhoven, The Netherlands) to characterize the ultrastructure of isolated cells.

## 3. Results

### 3.1. Morphology of Alveolar Basal Cells Cultured in DMEM Growth Medium

**Figure 1:**
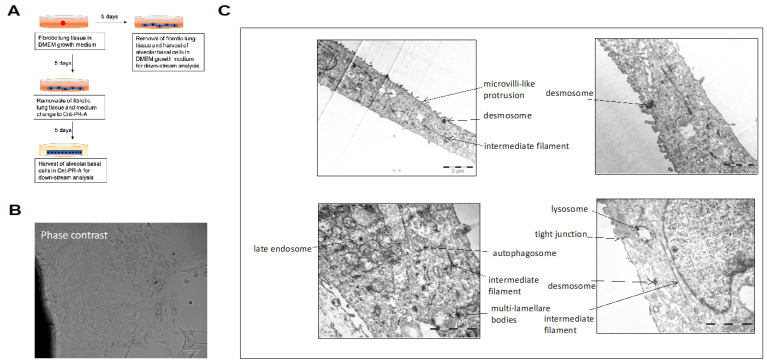
Morphology of alveolar basal cells cultured in DMEM growth medium. Experimental design of the study (**A**). Basal-like cells were cultured in DMEM growth medium for 5 days and then analyzed by (**B**) phase contrast microscopy, or (**C**) electron microscopy.

We previously reported the outgrowth of KRT17+/KRT5+ alveolar basal cells from peripheral fibrotic lung tissue [9]. The morphology of the alveolar basal cells cultured in DMEM growth medium for five days is shown in representative phase contrast (Figure 1A) and EM (Figure 1B) images. The cells display a flattened morphology showing a certain polarity. Microvilli-like structures were observed on their surface and desmosomes and tight junctions joined the cells. Multi-lamellate bodies were occasionally seen. Bundles of intermediate filaments were often found oriented along the axis of the cell and connected to the plaques of the desmosomes. Occasionally, late endosomes and autophagosomes were detected in the cells (Figure 1B). 

### 3.2. scRNA-Seq Analysis of Alveolar Basal Cells Cultured in DMEM Growth Medium

**Figure 2:**
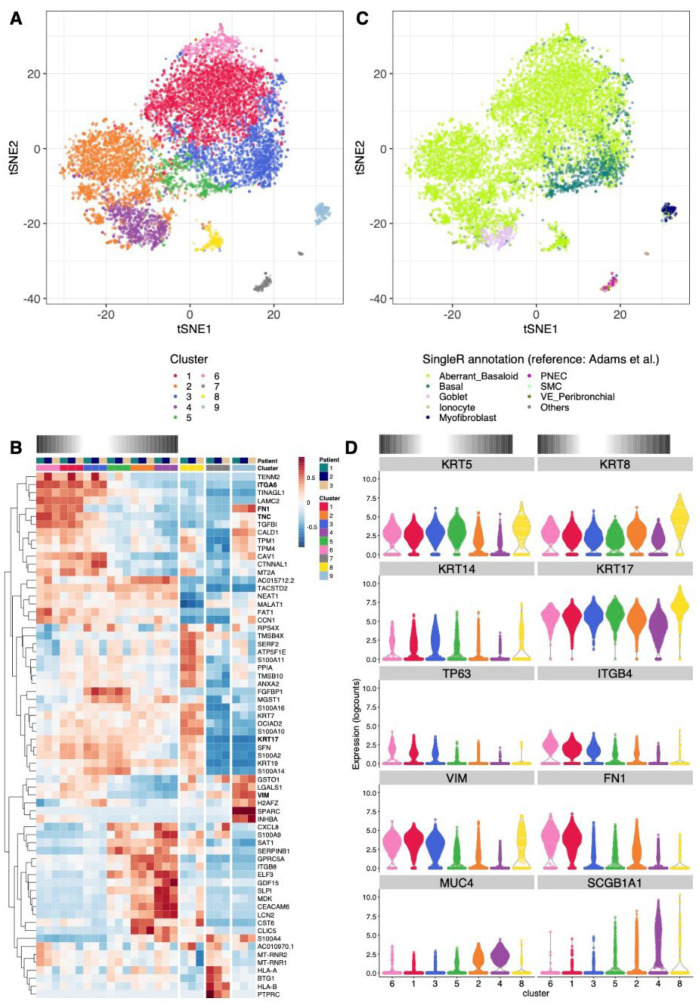
Clustering and cell type annotation of alveolar basal cells cultured in DMEM growth medium. (**A**) tSNE showing the separation of cells into nine clusters. (**B**) t-SNE showing the best matching cell type for each cell from the SingleR analysis using as reference the scRNA-seq dataset from Adams et al. [6]. Cell types represented by no more than 10 cells in our dataset were pooled into the category “Others”. Reference cell types originating from IPF or control patients are not distinguished in this figure, but see Supplement Figure S2A. (**C**) Relative expression across clusters and patients of the strongest cluster-specific genes. The log-normalized expression levels were averaged across cells from the same cluster and patient, and averaged values are then centered and scaled per gene. Clusters of epithelial cells are ordered along the presumed differentiation gradients described in the text, indicated with a gradient color bar on top of the heatmap. (**D**) Violin plot showing the log-normalized expression of basal cell marker KRT5, KRT17, KRT14, TP63, ITGB4, mesenchymal marker FN1 or VIM, secretory epithelial cell marker MUC4 or SCGB1A1 or transitional epithelial cell marker KRT8 across epithelial clusters. Only clusters of epithelial cells (i.e., without clusters 7 and 9) are shown, ordered along the presumed differentiation gradients described in the text, indicated with a gradient color bar on top of the panel.

To characterize this population of alveolar basal cells, we performed scRNA-seq using the 10× Genomics technology. The cells were obtained from lung tissue of three different IPF patients and were cultured in DMEM growth medium for five days [9]. After cell quality filtering and removal of patient-specific effects (Supplement Figure S1), the 8625 cells were grouped into nine clusters, encompassing between 2.1% and 31% of the cells (Figure 2A). The expression patterns of the top cluster-specific genes (full gene list available from Supplement Table S2) and the comparison of our dataset to publicly available scRNA-seq atlases of the human lung [6,7,31,35] allowed us to annotate these clusters (Figure 2B,C and Figure S2A–E). The majority of our cultured cells show transcriptomic similarity closest to cells derived from IPF patients and not to cells from control donors (Supplement Figure S2A,B). This suggests that the fibrotic signature of the cells is maintained in vitro.

The transcriptome of cells from clusters 1, 2, 6 and 8 matched best to an aberrant basaloid cell population reported by Adams et al. (Figure 2C and Figure S2A) [6] and Haberman et al. [7] (Supplement Figure S2B, coined “KRT5−/KRT17+” cells). Of note, this population is not present in the other references used (Reyfman et al. [35] and Travaglini et al. [31]; Supplement Figure S2C,D), so cells from these four clusters had closest transcriptomic similarity to the basal cells from these datasets. Similar to the described aberrant basaloid cells, cells from clusters 1 and 6 expressed the mesenchymal markers FN1 and VIM, along with the basal cell marker KRT17. The expression of the basal cell marker KRT5 was only slightly lower (Figure 2B,D and Figure S3A–C), while it was reported absent in the aberrant basaloid cell population (see Discussion).

The marker for transitional epithelial cells KRT8 was expressed in cells of all epithelial clusters, consistent with a transcriptomic proximity close to a population of KRT8+ alveolar differentiation intermediates described in mice (Supplement Figure S2F) [19].

A scRNA-seq reference dataset from organoids derived from cultured human AT2 cells undergoing trans-differentiation to basal cells in vitro [5] provided interesting insights into cells from the clusters 2 and 8, which matched closely to the alveolar-basal intermediates ABI1 and ABI2, respectively (Supplement Figure S2E). These cells expressed the mesenchymal markers VIM and FN1 at lower levels than clusters 1 and 6 and cells from cluster 2 expressed low levels of KRT5 (Figure 2D and Figure S3A,C).

From the remaining cells in our dataset, those from cluster 3 and 5 matched closely to basal cells or proliferating basal cells (Figure 2C and Figure S2A–E). They displayed a strong expression of KRT5 and KRT17 (Figure 2B,D and Figure S3A) as well as the basal cell markers KRT14, TP63, ITGB4 and ITGA6 (Figure 2B,D). The strong proliferation signal in cluster 3 was confirmed by the inferred cell cycle phase, the expression of MKI67 and a proliferative signature score gathering 11 markers [31] (Supplement Figure S4). 

Cells from cluster 4 matched best to more differentiated types of secretory epithelial cells, such as goblet or club cells (Figure 2C and Figure S2A–E). Accordingly, they expressed the secretory epithelial cell markers SCGB1A1 and MUC4, along with a reduced expression of KRT17 and KRT8, and a low expression of all other basal markers (KRT5, TP63, KRT14, ITGB4 and ITGA6; Figure 2B,D and Figure S3A,B,D).

Finally, cells from cluster 9 clearly matched to mesenchymal cells, with notably the strongest expression of VIM and FN1 (Figure 2B). The identity of cells from cluster 7 was less clear. Depending on the reference dataset used, they matched best to endothelial, epithelial, or immune subtypes (Supplement Figure S2A–E). The high expression of MHC class I and CD45 (PTPRC) genes (Figure 2B) corroborated this.

In further analyses we excluded cells from non-epithelial clusters 7 and 9 to focus on the bulk of epithelial cells.

**Figure 3:**
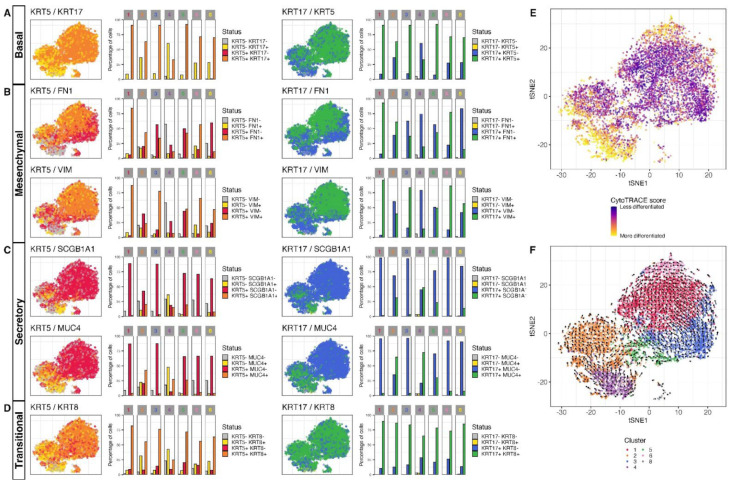
Co-expression of specific marker and differentiation states and trajectory in alveolar basal cells cultured in DMEM growth medium. tSNE showing co-expression patterns of (**A**) KRT5 and KRT17 with (**B**) FN1, VIM, (**C**) SCGB1A1, MUC4 or (**D**) KRT8. A log-normalized expression level of 0.5 was used to call genes expressed or not expressed. The relative proportion of cells expressing/co-expressing the different markers across clusters is shown in bar plots. (**E**) tSNE showing the CytoTRACE differentiation state scores across cells. (**F**) tSNE overlaid with the embedded velocity vectors of the RNA velocity showing the differentiation trajectories inferred from comparison of spliced and unspliced signal across genes in single cells. A grid-based approach is used to summarize the per-cell vectors into local representatives for effective visualization.

The expression and co-expression patterns of these markers (Figure 3A–D) indicated a differentiation gradient from basal cells (KRT5^high^/KRT17^high^ cells from clusters 3 and 5) to aberrant basaloid-like cells (KRT5^intermediate^/KRT17^high^/FN1^high^/VIM^high^ cells from clusters 1 and 6) on one side, and to secretory epithelial-like cells on the other (KRT5^low^/KRT17^low^/MUC4^high^/SCGB1A1^high^ cells of cluster 4). Both these gradients were supported by a CytoTRACE analysis, a computational method inferring the differentiation state of cells by using the number of detectably expressed genes as a determinant of developmental potential [34] (Figure 3E). An RNA velocity analysis, using the signal of spliced and unspliced mRNAs to infer the short-term dynamics in gene expression state across cells [40,41], confirmed a clear trajectory from cluster 3 to 1 and 6. There was some path from basal cells of cluster 5 towards secretory epithelial-like cells of cluster 4, but this came along with another strong trajectory starting from cluster 4 towards cluster 2 (Figure 3F).

### 3.3. Alveolar Basal Cell Differentiation towards Secretory Epithelial- or Aberrant Basaloid-like Cells Is Reversible

**Figure 4:**
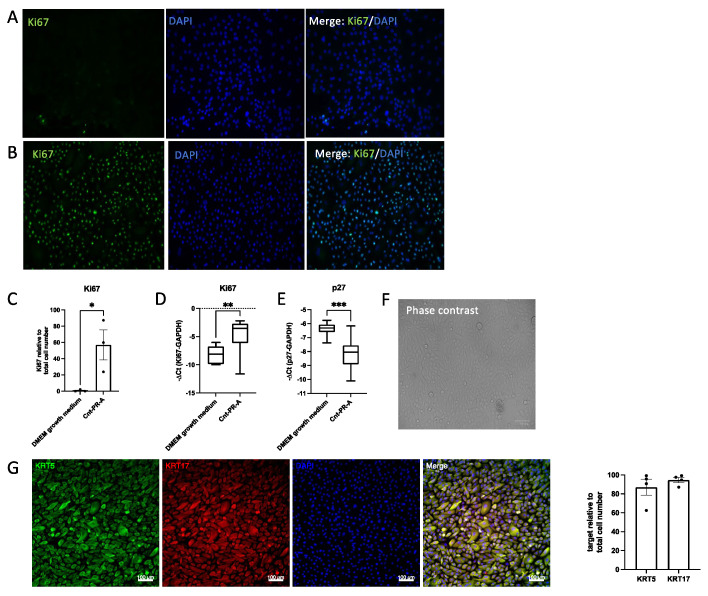
Alveolar basal cell proliferation in DMEM growth medium versus Cnt-PR-A. Representative Ki67 immunofluorescence (IF) stainings (*n* = 3) of cells (**A**) cultured in DMEM growth medium for 5 days or (**B**) after medium change to Cnt-PR-A and culture for an additional 5 days. (**C**) Quantification of Ki67 IF stainings relative to total cell number (DAPI) in DMEM growth medium and Cnt-PR-A. Dots (•) in the bar chart represent the individual datapoints generated from cell stainings of three different patients. * indicates *p* < 0.05 (unpaired student’s *t*-test). (**D**) Ki67 or (**E**) p27 RNA expression comparing both culture conditions (*n* = 12). TaqMan PCR data are expressed as −ΔCt. ** indicates *p* = 0.0017, *** indicates *p* = 0.0002 (unpaired student’s *t*-test). (**F**) Representative phase contrast image of alveolar basal cells in Cnt-PR-A, (**G**) Representative immunofluorescence images (*n* = 4) and their quantification relative to the total cell number (DAPI) showing the expression of KRT17 and KRT5 in cells cultured in Cnt-PR-A. Dots (•) in the bar chart represent the individual datapoints generated from cell stainings of four different patients.

Keeping cultured alveolar basal cells in DMEM growth medium for more than five days usually resulted in no further cell proliferation, even when the medium was regularly exchanged with fresh growth medium (Supplement Figure S5). Accordingly, after five days in DMEM growth medium very few cells were positive for the proliferation marker Ki67 (Figure 4A). On the contrary, when the medium was changed to an epithelial-specific growth medium (Cnt-PR-A), cells showed robust proliferation and an increased number of Ki67 positive cells (Figure 4B). This was confirmed by TaqMan PCR, showing higher levels of Ki67 RNA expression (Figure 4C) and lower levels of the cell cycle inhibitor p27 (Figure 4E) in cells cultured in Cnt-PR-A compared to cells cultured in DMEM growth medium. Furthermore, in Cnt-PR-A the cell morphology changed from flattened (Figure 1B) to that of cuboidal epithelial cells as displayed in a representative phase contrast image (Figure 4F). Alveolar basal cells showed positivity for the basal cell markers KRT17 and KRT5 in DMEM growth medium [9] and in Cnt-PR-A (Figure 4G).

**Figure 5:**
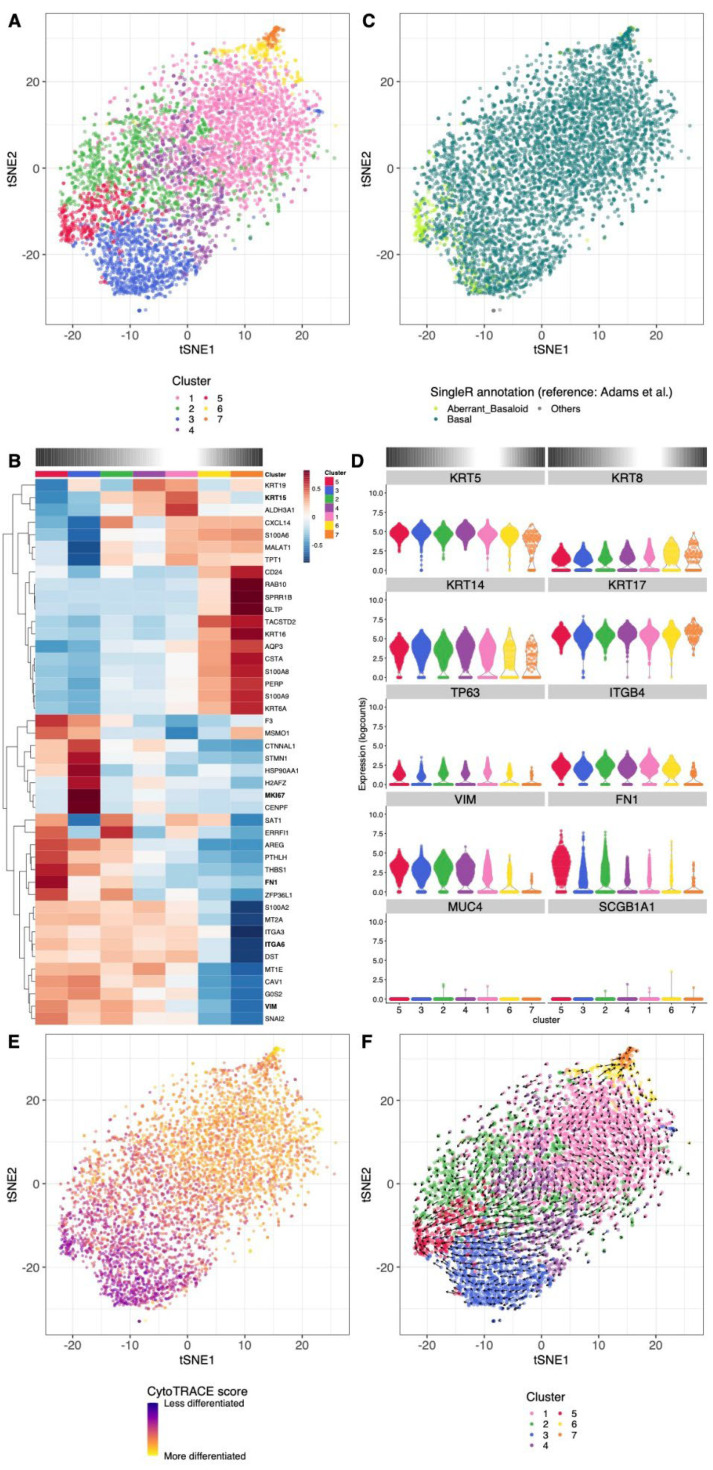
Clustering and cell type annotation of alveolar basal cells cultured in Cnt-PR-A. (**A**) tSNE showing the separation of the cells into seven clusters. (**B**) t-SNE showing the best matching cell type for each cell, similar to Figure 2B. (**C**) Relative expression across clusters of the strongest cluster-specific genes in the dataset. Clusters are ordered to reflect the presumed differentiation gradients described in the text, indicated with a gradient color bar on top of the heatmap. (**D**) Violin plot showing the log-normalized expression of basal cell marker KRT5, KRT17, KRT14, TP63, ITGB4, mesenchymal marker FN1 or VIM, secretory epithelial cell marker MUC4 or SCGB1A1 or transitional epithelial cell marker KRT8 across clusters. Clusters are ordered to reflect the presumed differentiation gradients described in the text, indicated with a gradient color bar on top of the panel. (**E**) tSNE showing the CytoTRACE differentiation state scores across cells. (**F**) tSNE overlaid with the embedded velocity vectors of the RNA velocity analysis, similar to Figure 3F.

To characterize the effect of this medium change at the transcriptomic level, we performed scRNA-seq on cells derived from an IPF patient that were first cultured in DMEM growth medium for five days and harvested after five more days in medium changed to Cnt-PR-A. After cell quality filtering (Supplement Figure S6), the 5280 cells were grouped into seven clusters, each encompassing between 1.6% and 37% of the cells (Figure 5A). The relative expression of the top cluster-specific genes is shown in Figure 5B (full gene list available from Supplement Table S3). The reference-based annotation of our dataset this time yielded a simpler picture with the vast majority of the cells best matching the transcriptome of basal cells (Figure 5C and Figure S7A–E). Of note, our cultured cells again showed closest transcriptional similarity to cells from IPF patients relative to those of control patients (Supplement Figure S7A,B). This suggests that their fibrotic signature is maintained in vitro even after changing conditions and prolonged culture of the cells.

The shift in the cell transcriptome upon transfer to Cnt-PR-A medium was reflected by the up-regulation of the basal cell markers KRT17 and KRT5, the loss of the secretory markers SCGB1A1 and MUC4, and the reduction in expression of the mesenchymal markers FN1 and VIM, and the transitional epithelial cell marker KRT8 (Figure 5B,D and Figure S8A–D). However, the shift from aberrant basaloid-like to basal cells did not seem fully complete, with cells from cluster 5 still closely matching the aberrant basaloid transcriptome (Figure 5C and Figure S7A,B).

A single cluster (cluster 3) was distinctive with a strong proliferation signal and expression of the marker MKI67 (Figure 2B and Figure S9). Using the proliferative signature score, we could confirm an increase in cell proliferation in Cnt-PR-A medium (11.9% of the cells proliferating) relative to DMEM growth medium (7.4% of the cells proliferating). 

Interestingly a gradient in expression of different markers was still present, leading us to investigate potential differentiation trajectories. CytoTRACE analysis confirmed a differentiation gradient originating from clusters 3 and 5 towards clusters 6 and 7 (Figure 5E). An RNA velocity analysis (Figure 5F) indeed confirmed a trajectory towards more differentiated cells of clusters 6 and 7, but surprisingly there was also a clear trajectory towards the aberrant basaloid-like cells (cluster 5). This trajectory path towards a less differentiated state would require further investigation.

**Figure 6:**
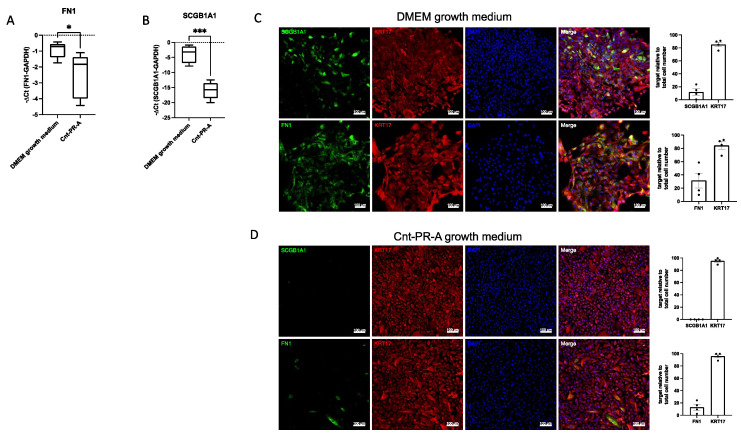
Validation of scRNA data by TaqMan PCR and immunofluorescence stainings. (**A**) FN1 or (**B**) SCGB1A1 RNA expression in cells cultured in DMEM growth medium or Cnt-PR-A (*n* = 5). Data are expressed as −ΔCt. * indicates *p* < 0.05, *** indicates *p* = 0.0002 (unpaired student’s *t*-test). Immunofluorescence images (*n* = 4) showing the co-expression of KRT17 with FN1 or SCGB1A1 in cells cultured in DMEM growth medium (**C**,**D**) in Cnt-PR-A and their respective quantification relative to the total cell number (DAPI). Dots (•) in the bar charts represent the individual datapoints generated from cell stainings of four different patients.

TaqMan PCR and immunofluorescence analysis confirmed decreased expression of the mesenchymal cell marker FN1 (Figure 6A,C) and the secretory epithelial cell marker SCGB1A1 (Figure 6B,C) in cells cultured in Cnt-PR-A compared to cells cultured in DMEM growth medium (Figure 6A,B,D), validating our scRNA-seq data.

## 4. Discussion

In this study, we analyzed cultured alveolar basal cells under different cell culture conditions. In DMEM growth medium, alveolar basal cells readily grew from peripheral fibrotic lung tissue, as described in our previous studies [9]. The cultured cells displayed a commonly observed flattened morphology, promoting epithelial cell differentiation [42,43]. scRNA-seq analysis demonstrated a differentiation gradient from basal to secretory epithelial-like cells, reflected by reduced expression of the basal cell marker KRT5 and the appearance of secretory epithelial cell markers in the more differentiated cell fraction. Airway basal cells have the ability to differentiate into secretory epithelial cells [4], suggesting that alveolar basal cells possess similar differentiation capacities. In the peripheral IPF lung, basal cells line pathological honeycomb cysts [44] which contain a bronchiolar-like epithelium [44], suggesting that differentiation of alveolar basal cells towards secretory epithelial cells also occurs in vivo. In our culture model, we observed only reversible differentiation towards secretory epithelial-like cells. Specific culture conditions, such as air-liquid-interface (ALI) [45] or organoid culture [46], may be required for the full differentiation to occur.

Surprisingly, a large fraction of cultured alveolar basal cells showed transcriptomic similarities closest to the recently described KRT17+/KRT5− aberrant basaloid cells [6,7]. A direct culture and expansion of the aberrant basaloid cells present in the fibrotic lung tissue with our culture method seems unlikely, as these cells are only present at very low numbers and specifically located on the surface of fibroblastic foci [6,7]. In an earlier study, we demonstrated that KRT17+/KRT5+ basal cells were abundant in peripheral IPF lung tissue and that individual tissue pieces that showed outgrowth of alveolar basal cells contained KRT17+/KRT5+ basal cells [9]. Therefore, KRT17+/KRT5+ alveolar basal cells cultured in DMEM growth medium likely differentiate towards aberrant basaloid-like cells in vitro, a pattern confirmed in our scRNA-seq dataset by a clear differentiation gradient originating from a cluster of proliferating basal cells towards aberrant basaloid-like cells. However, a main characteristic feature of aberrant basaloid cells is the absence of KRT5 [6,7]. Aberrant basaloid-like cells in our culture still express KRT5, suggesting that these cells could be primed but not fully differentiated towards aberrant basaloid cells. A recent study demonstrated that human AT2 cells have the capacity to trans-differentiate to basal cells, via SFTPC+/KRT17+/KRT5− alveolar-basal-intermediate (ABI)1 and SFTPC−/KRT17+/KRT5− ABI2 cells [5]. Another study described the occurrence of KRT8+ alveolar differentiation intermediates (ADI) during AT2 to AT1 differentiation [19]. Interestingly, ABI1, ABI2 and KRT8+ ADI cells showed some transcriptional similarity to aberrant basaloid cells [5,19]. Our dataset included cells with similarity to these ABI1, ABI2 and KRT8+ ADI cells, indicating that they might have the capacity to trans-differentiate towards AT2 cells, as was previously demonstrated in mice [12,13]. Supporting this, our cultured cell population contains cells showing some typical characteristics of AT cells (microvilli-like structures and multi-lamellar bodies) on EM images. However, this assumption remains highly speculative and needs to be proven in future studies.

Importantly, we here show that differentiation of cultured alveolar basal cells towards secretory epithelial-like cells or aberrant basaloid-like cells is largely reversible by changing the cell culture medium. This suggests that the cell differentiation capacity is largely influenced by their specific microenvironment. A previous study proposed that the IPF microenvironment may initiate an abnormal differentiation program in resident AT cells, and that this may be responsible for the appearance of atypical epithelial cells in the peripheral IPF lung [47]. Furthermore, in bleomycin-injured animal lungs, bronchial epithelial stem cells were shown to contribute either to pathological bronchiolization or to alveolar regeneration depending on the cells microenvironment and subsequent activation of specific signaling pathways [13].

## 5. Conclusions

In this study, we cultured primary alveolar basal cells from peripheral lung tissue of IPF patients, which provide a valuable model for IPF research. The low number of patients included in the study limits the robustness of our results and we are aware that alveolar basal cells cultured in vitro may not fully reflect the cells characteristics and capacities in vivo. However, we provide evidence that while cultured primary human alveolar basal cells maintain their fibrotic signature in vitro, they have the potential to undergo reversible differentiation towards secretory epithelial or aberrant basaloid-like cells. This seems highly dependent on their microenvironment. Determining specific factors that induce or reverse these differentiation paths may help to define novel drug targets for IPF therapy.

## Data Availability

The processed scRNA-sequencing dataset (the accession is GSE198153) can be viewed on the gene expression omnibus database (GEO).

## Supplementary Materials

The following supporting information can be downloaded at: https://www.mdpi.com/article/10.3390/cells11111820/s1, Supplement Figure S1: tSNE showing the distribution of cells cultured in DMEM growth medium from patient 1, 2, and 3; the percentage of scRNA-seq reads originating from mitochondrial genes, and the number of detected genes across cell; Supplement Figure S2: tSNE showing the best matching cell type from the *SingleR* analysis for each cell cultured in DMEM growth medium using different datasets as reference; Supplement Figure S3: tSNE showing the log-normalized expression of different marker genes in cells cultured in DMEM growth medium; Supplement Figure S4: tSNE showing the inferred cell-cycle phase, the log-normalized expression of MKI67, and the proliferative signature score in cells cultured in DMEM growth medium; Supplement Figure S5: Cell counts of alveolar basal cells cultured in DMEM growth medium at different time points; Supplement Figure S6: tSNE of cells cultured in Cnt-PR-A showing the percentage of scRNA-seq reads originating from mitochondrial genes and the number of detected genes across cell; Supplement Figure S7: tSNE showing the best matching cell type from the *SingleR* analysis for each cell cultured in Cnt-PR-A; Supplement Figure S8: tSNE showing the log-normalized expression of different marker genes in cells cultured in Cnt-PR-A; Supplement Figure S9: tSNE showing the inferred cell-cycle phase, the log-normalized expression of MKI67, and the proliferative signature score in cells cultured in Cnt-PR-A. Supplement Table S1: Materials; Supplement Table S2: Cluster specific genes in cells cultured in DMEM growth medium; Supplement Table S3: Cluster specific genes in cells cultured in Cnt-PR-A.
