# Supplementary material for: Alveolar Basal Cells Differentiate towards Secretory Epithelial- and Aberrant Basaloid-like Cells In Vitro
Source: Cells. 2022 Jun 2;11(11):1820. doi: 10.3390/cells11111820 (PMC9180703; doi:10.3390/cells11111820)
Supplement: Supplementary file 1 [file cells-11-01820-s001.zip › New supplement figure S2.pdf]

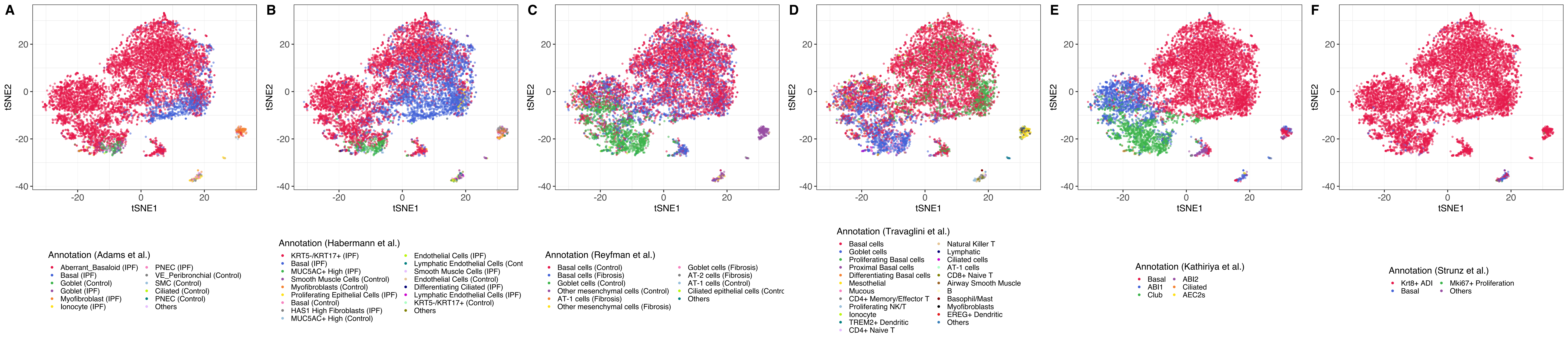
A
B
C
D
E
F
tSNE1
tSNE2
20
0
-20
-40
-20
0
20
Annotation (Adams et al.)
Aberrant_Basaloid (IPF)
Basal (IPF)
Goblet (Control)
Goblet (IPF)
Myofibroblast (IPF)
Ionocyte (IPF)
PNEC (IPF)
VE_Peribronchial (Control)
SMC (Control)
Ciliated (Control)
PNEC (Control)
Others
Annotation (Habermann et al.)
KRT5-/KRT17+ (IPF)
Basal (IPF)
MUC5AC+ High (IPF)
Smooth Muscle Cells (Control)
Myofibroblasts (Control)
Proliferating Epithelial Cells (IPF)
Basal (Control)
HAS1 High Fibroblasts (IPF)
MUC5AC+ High (Control)
Endothelial Cells (IPF)
Lymphatic Endothelial Cells (Cont
Smooth Muscle Cells (IPF)
Endothelial Cells (Control)
Differentiating Ciliated (IPF)
Lymphatic Endothelial Cells (IPF)
KRT5-/KRT17+ (Control)
Others
Annotation (Reyfman et al.)
Basal cells (Control)
Basal cells (Fibrosis)
Goblet cells (Control)
Other mesenchymal cells (Control)
AT-1 cells (Fibrosis)
Other mesenchymal cells (Fibrosis)
Goblet cells (Fibrosis)
AT-2 cells (Fibrosis)
AT-1 cells (Control)
Ciliated epithelial cells (Contrc
Others
Annotation (Travaglini et al.)
Basal cells
Goblet cells
Proliferating Basal cells
Proximal Basal cells
Differentiating Basal cells
Mesothelial
Mucous
CD4+ Memory/Effector T
Proliferating NK/T
Ionocyte
TREM2+ Dendritic
CD4+ Naive T
Natural Killer T
Lymphatic
Ciliated cells
AT-1 cells
CD8+ Naive T
Airway Smooth Muscle
B
Basophil/Mast
Myofibroblasts
EREG+ Dendritic
Others
Annotation (Kathiriya et al.)
Basal
ABI1
Club
ABI2
Ciliated
AEC2s
Annotation (Strunz et al.)
Krt8+ ADI
Basal
Mki67+ Proliferation
Others
