# Supplementary material for: Alveolar Basal Cells Differentiate towards Secretory Epithelial- and Aberrant Basaloid-like Cells In Vitro
Source: Cells. 2022 Jun 2;11(11):1820. doi: 10.3390/cells11111820 (PMC9180703; doi:10.3390/cells11111820)
Supplement: Supplementary file 1 [file cells-11-01820-s001.zip › Supplement Table S1.pdf]

Supplement Table 1: Materials

Product	Company	Catalog Number	Country
DMEM	Thermo Fisher Scientific	41965-039	Waltham, MA, USA
FCS	Thermo Fisher Scientific	16140-063	Waltham, MA, USA
HEPES	Thermo Fisher Scientific	15630-080	Waltham, MA, USA
MEM-vitamin mix (100x)	Thermo Fisher Scientific	25030-081	Waltham, MA, USA
Antibiotic-Antimycotic (100x)	Thermo Fisher Scientific	15240-062	Waltham, MA, USA
Sodium pyruvate (100x)	Thermo Fisher Scientific	11360-039	Waltham, MA, USA
Cnt-PR-A	CELLnTEC		Bern, Switzerland
Quick-RNA MiniPrep Kit	ZymoResearch	R1050	Orange, CA, USA
GAPDH TaqMan® Gene Expression Assay	Thermo Fisher Scientific	Hs03929097_g1	Waltham, MA, USA
FN1 TaqMan® Gene Expression Assay	Thermo Fisher Scientific	Hs01549976_m1	Waltham, MA, USA
SCGB1A1 TaqMan® Gene Expression Assay	Thermo Fisher Scientific	Hs00171092_m1	Waltham, MA, USA
TaqMan™ Universal PCR Master Mix, no AmpErase UNG	Thermo Fisher Scientific	4324018	Waltham, MA, USA
MKI67 TaqMan® Gene Expression Assay	Thermo Fisher Scientific	Hs01032433_m1	Waltham, MA, USA
CDKN1B (p27) TaqMan® Gene Expression Assay	Thermo Fisher Scientific	Hs00153277_m1	Waltham, MA, USA
DAPI	Thermo Fisher Scientific	62248	Waltham, MA, USA
Rat-Ki67-antibody, FITC	Thermo Fisher Scientific	11-5698-82	Waltham, MA, USA
Mouse-KRT17 antibody	Thermo Fisher Scientific	MA1-06325	Waltham, MA, USA
Rabbit-KRT17 antibody	Thermo Fisher Scientific	PA5-27949	Waltham, MA, USA
Mouse-KRT5 antibody	Thermo Fisher Scientific	MA5-12596	Waltham, MA, USA
Mouse-FN1 antibody	Thermo Fisher Scientific	MA5-11981	Waltham, MA, USA
Rat-SCGB1A1 antibody	RnD System	394324	Abingdon, UK
Alexa 488 donkey anti-rat	Thermo Fisher Scientific	21208	Waltham, MA, USA
Alexa 647 Goat anti-mouse	Thermo Fisher Scientific	A12235	Waltham, MA, USA
Alexa 488 Donkey anti-mouse	Thermo Fisher Scientific	A21202	Waltham, MA, USA

Alexa 555 goat anti-rabbit	Thermo Fisher Scientific	#A21428	Waltham, MA, USA
LIVE/DEAD^{TM} staining kit	Thermo Fisher Scientific	L10119	Waltham, MA, USA
