# Supplementary material for: Alveolar Basal Cells Differentiate towards Secretory Epithelial- and Aberrant Basaloid-like Cells In Vitro
Source: Cells. 2022 Jun 2;11(11):1820. doi: 10.3390/cells11111820 (PMC9180703; doi:10.3390/cells11111820)
Supplement: Supplementary file 1 [file cells-11-01820-s001.zip › cells-1674384 - Supplementary materials .pdf]

Supplementary materials

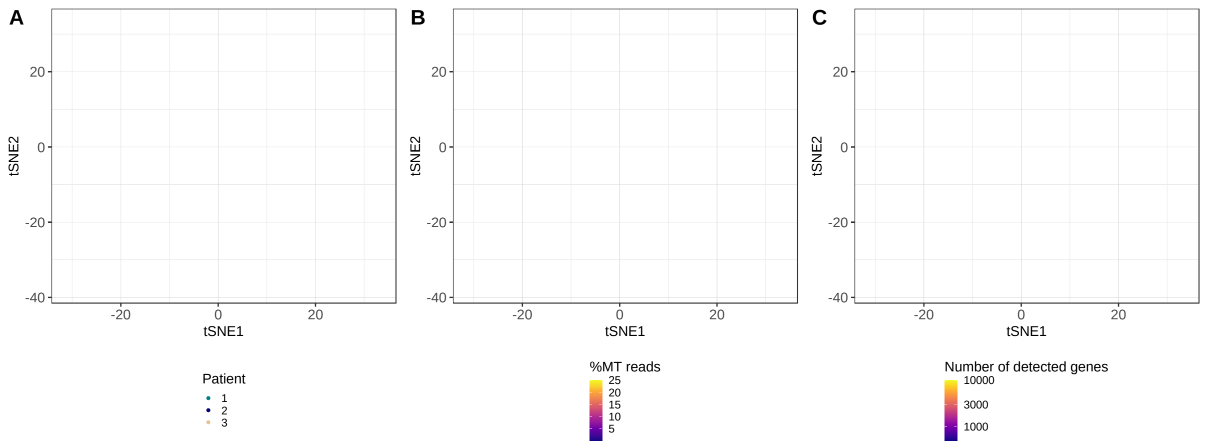

Supplement figure S1: A) tSNE of the retained cells from the dataset of cells cultured in DMEM growth medium, showing the distribution of cells from patient 1 (green), patient 2 (blue) and patient 3 (beige); B) the percentage of scRNA-seq reads originating from mitochondrial genes; and C) the number of detected genes across cell (number of genes with at least one UMI count, on a log-scale).

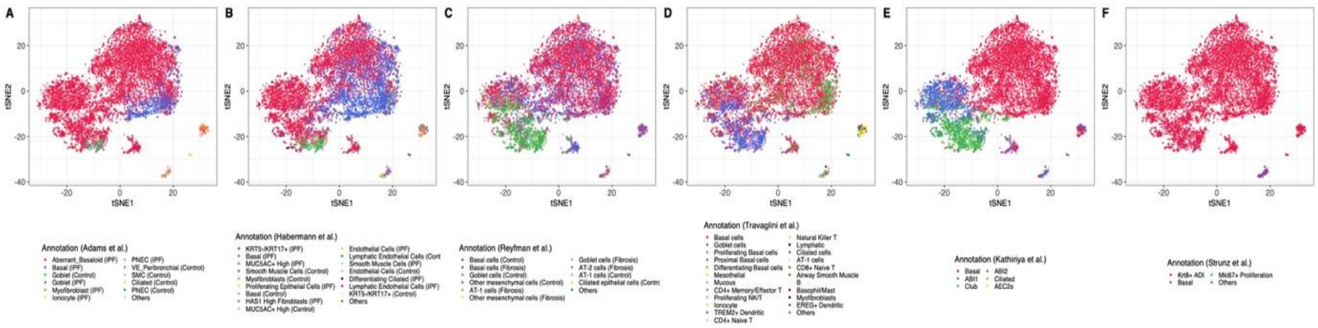

Supplement figure S2: tSNE showing the best matching cell type from the *SingleR* analysis for each cell cultured in DMEM growth medium A) using as reference the dataset from Adams et al. [1], B) using as reference the dataset from Habermann et al. [2] C), using as reference the dataset from Reyfmann et al. [3], reannotated by us (see Methods), D) using as reference the dataset from Travaglini et al. [4], E) using as reference the dataset from Kathiriya et al. [5], and F) using as reference the dataset from Strunz et al. [6]. Cell types represented by no more than 5 cells in our dataset were pooled into the category “Others”. Cell types are ordered according to their abundance in our dataset.

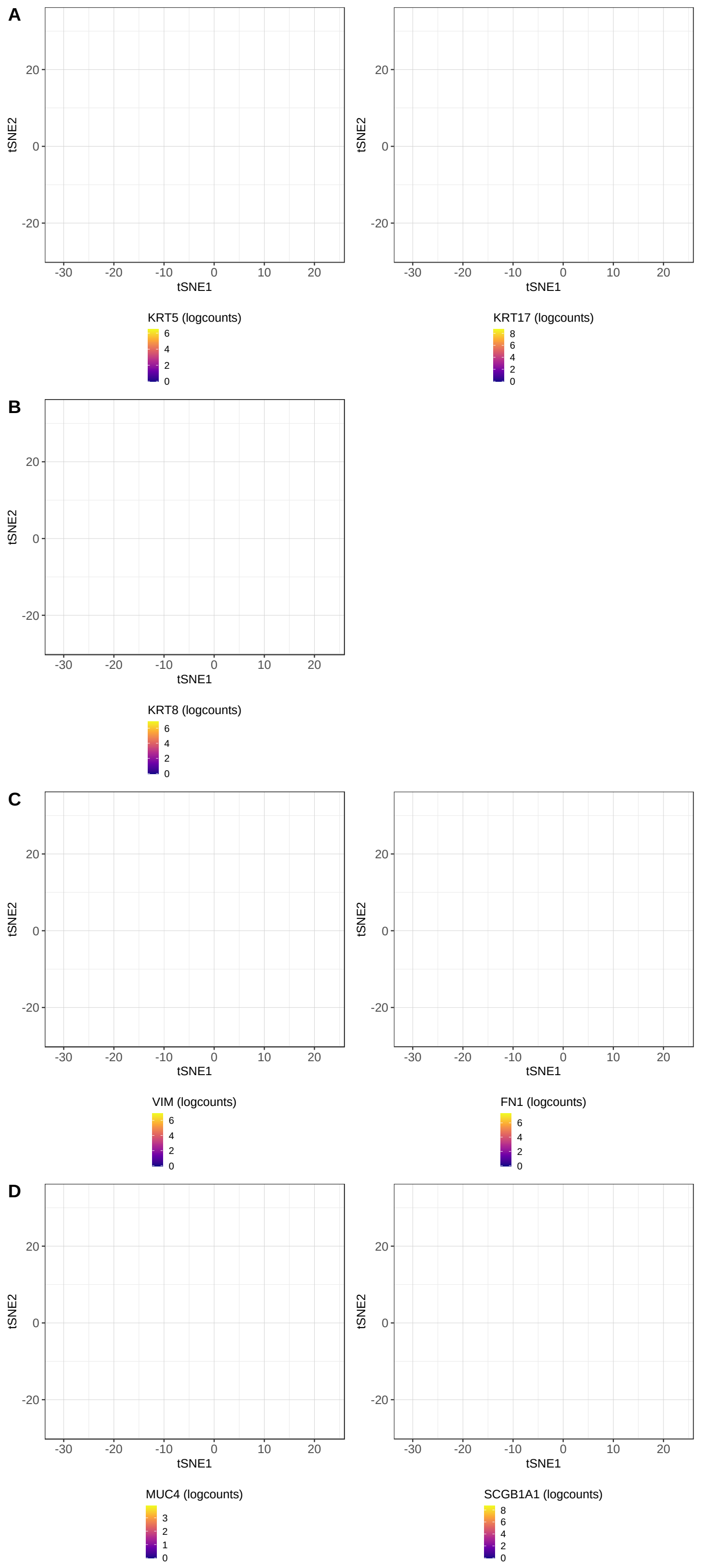

Supplement figure S3: tSNE showing the log-normalized expression of A) basal cell marker KRT17 or KRT5, B) transitional epithelial cell marker KRT8, C) mesenchymal marker FN1 or VIM, or D) secretory epithelial cell marker MUC4 or SCGB1A1 in cells cultured in DMEM growth medium.

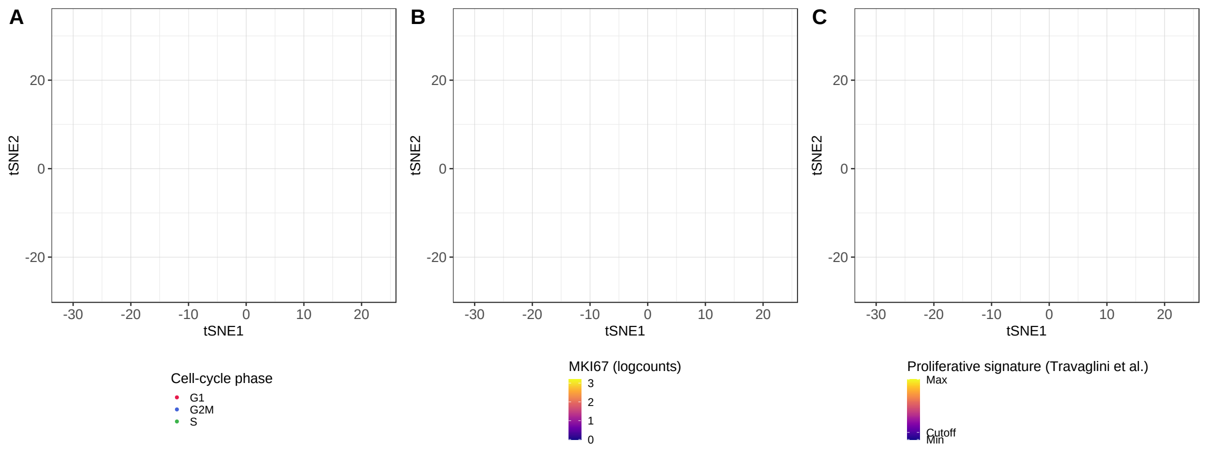

Supplement figure S4: tSNE showing the A) inferred cell-cycle phase B) the log-normalized expression of the MKI67 marker, and C) the proliferative signature score from Travaglini et al. [4] based on 11 markers in cells cultured in DMEM growth medium. The cutoff used to quantify the amount of proliferating cells is displayed in the legend color bar.

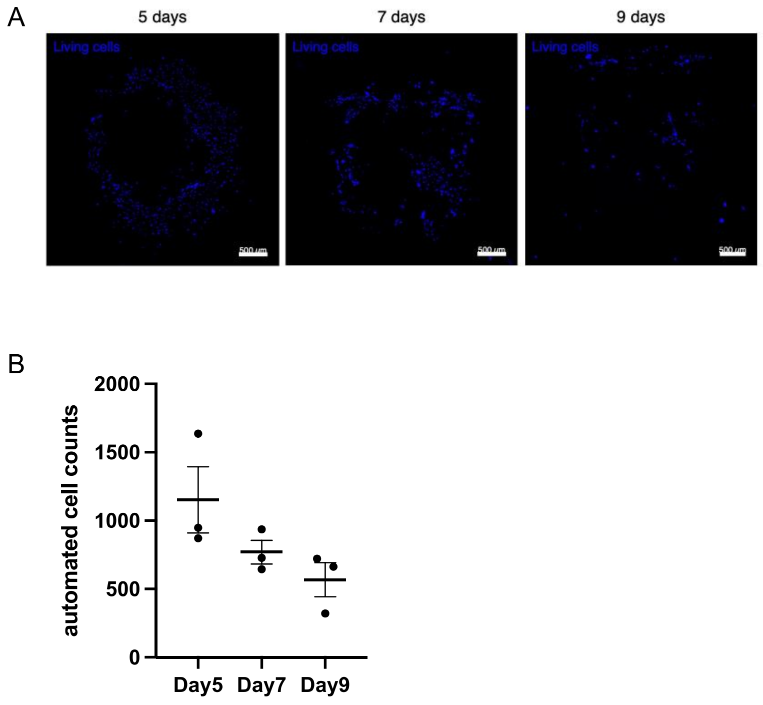

Supplement figure S5: A) Representative immunofluorescence image (n=3) of alveolar basal cells stained with live cell DAPI and B) their automated cell counts at day 5, 7 and 9 in DMEM growth medium.

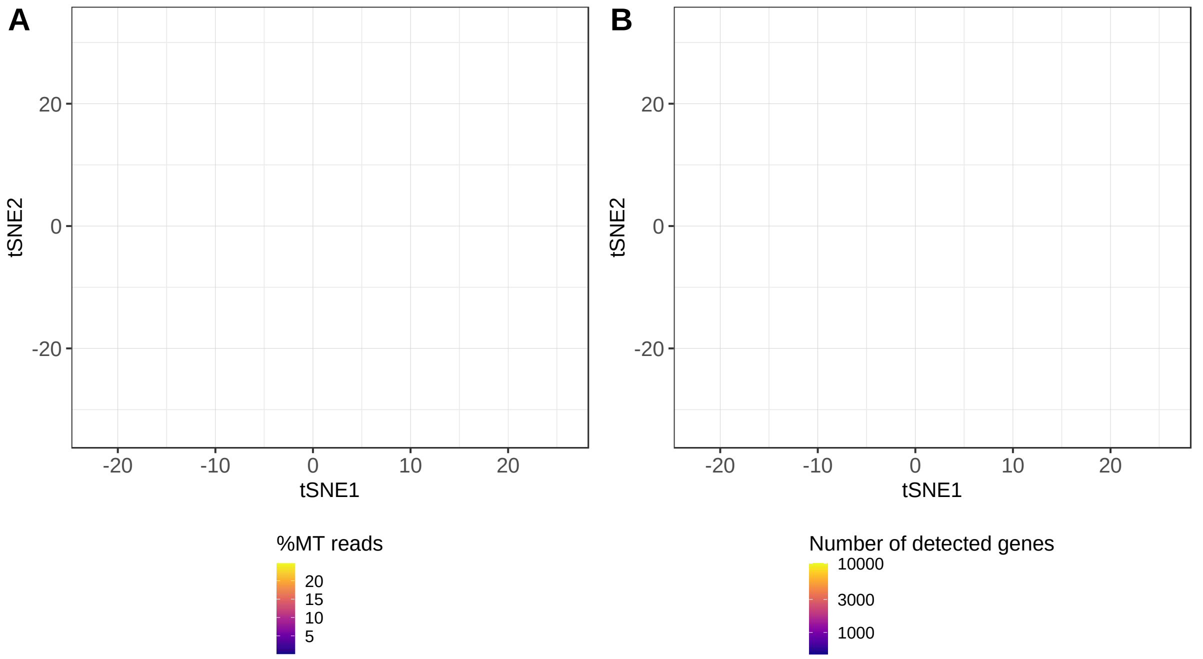

Supplement figure S6: A) tSNE of the retained cells from the dataset of cells cultured in Cnt-PR-A, showing the A) the percentage of scRNA-seq reads originating from mitochondrial genes and B) the number of detected genes across cell (number of genes with at least one UMI count, on a log-scale).

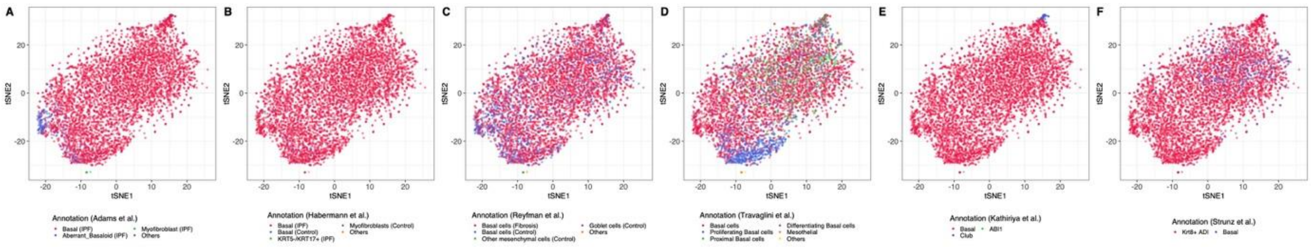

Supplement figure S7: tSNE showing the best matching cell type from the *SingleR* analysis for each cell cultured in Cnt-PR-A, similar to Supplement figure 2.

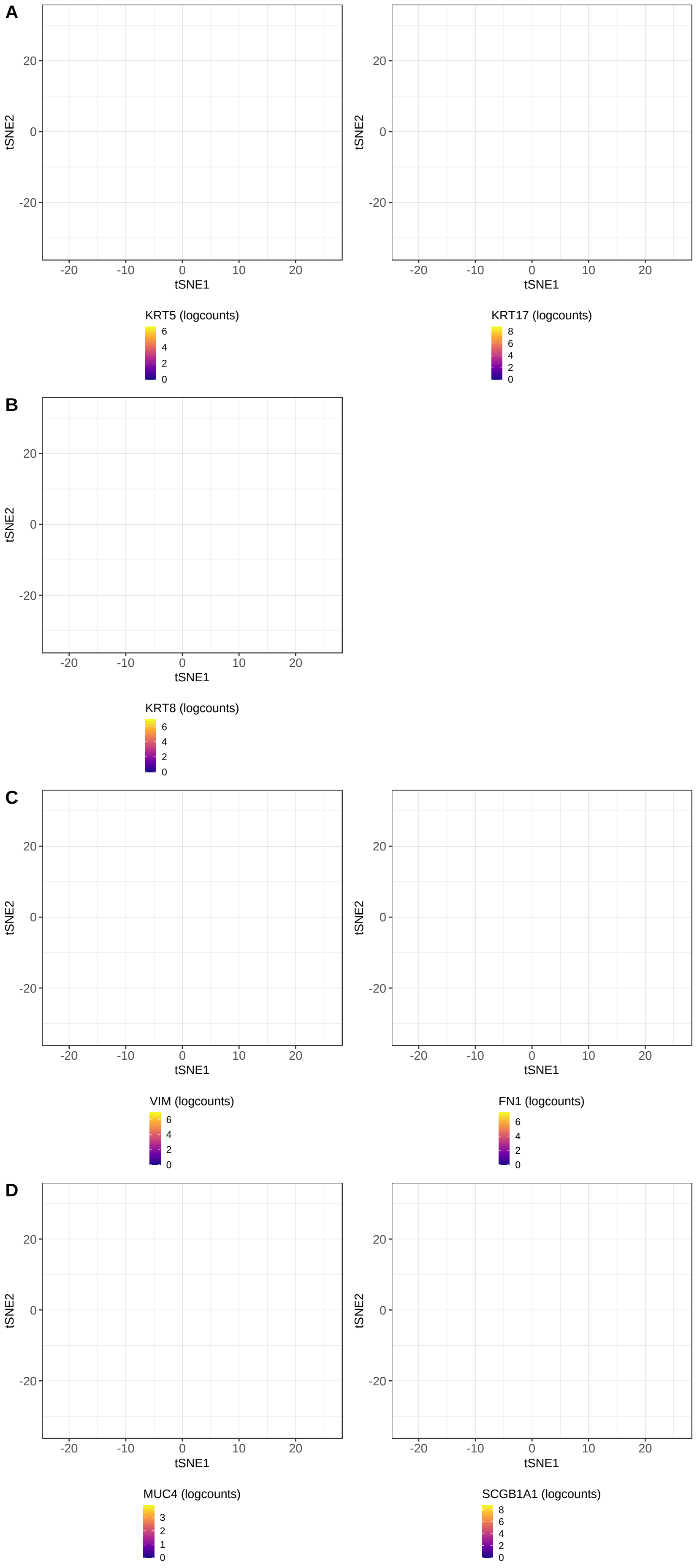
A
B
C
D
tSNE1
tSNE2
-20
-10
0
10
20
KRT5 (logcounts)
KRT17 (logcounts)
KRT8 (logcounts)
VIM (logcounts)
FN1 (logcounts)
MUC4 (logcounts)
SCGB1A1 (logcounts)

Supplement figure S8: tSNE showing the log-normalized expression of A) basal cell marker KRT17 or KRT5, B) transitional epithelial cell marker KRT8, C) mesenchymal marker FN1 or VIM, or D) secretory epithelial cell marker MUC4 or SCGB1A1 in cells cultured in Cnt-PR-A. The color scale used matches the color scale used in Supplement figure 3.

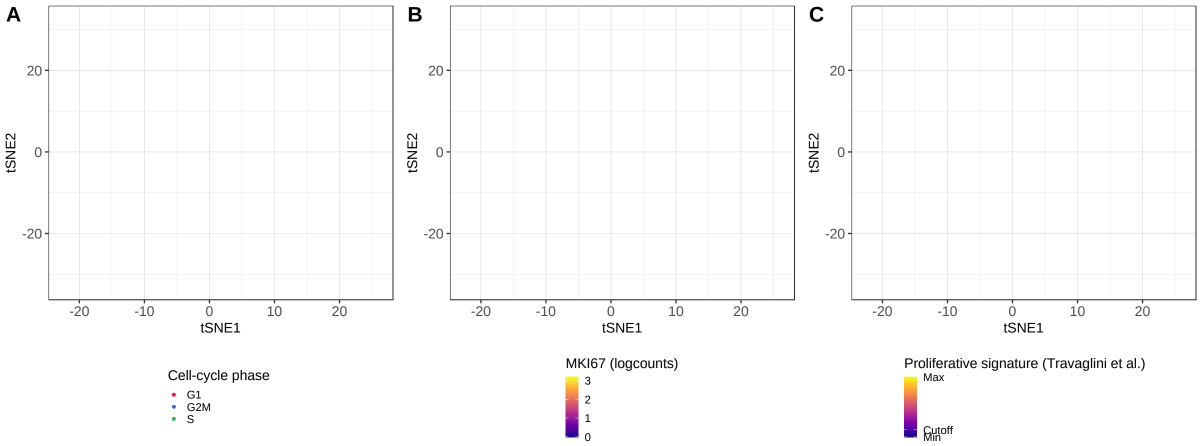

Supplement figure S9: tSNE showing the A) inferred cell-cycle phase B) the log-normalized expression of the MKI67 marker, and C) the proliferative signature score in cells cultured in Cnt-PR-A. The cutoff used to quantify the amount of proliferating cells is displayed in the legend color bar. The color scales used match the color scale used in Supplement figure 4.

Supplement Table 1: Materials

Product	Company	Catalog Number	Country
DMEM	Thermo Fisher Scientific	41965-039	Waltham, MA, USA
FCS	Thermo Fisher Scientific	16140-063	Waltham, MA, USA
HEPES	Thermo Fisher Scientific	15630-080	Waltham, MA, USA
MEM-vitamin mix (100x)	Thermo Fisher Scientific	25030-081	Waltham, MA, USA
Antibiotic-Antimycotic (100x)	Thermo Fisher Scientific	15240-062	Waltham, MA, USA
Sodium pyruvate (100x)	Thermo Fisher Scientific	11360-039	Waltham, MA, USA
Cnt-PR-A	CELLnTEC		Bern, Switzerland
Quick-RNA MiniPrep Kit	ZymoResearch	R1050	Orange, CA, USA
GAPDH TaqMan® Gene Expression Assay	Thermo Fisher Scientific	Hs03929097_g1	Waltham, MA, USA
FN1 TaqMan® Gene Expression Assay	Thermo Fisher Scientific	Hs01549976_m1	Waltham, MA, USA
SCGB1A1 TaqMan® Gene Expression Assay	Thermo Fisher Scientific	Hs00171092_m1	Waltham, MA, USA
TaqMan™ Universal PCR Master Mix, no AmpErase UNG	Thermo Fisher Scientific	4324018	Waltham, MA, USA
MKI67 TaqMan® Gene Expression Assay	Thermo Fisher Scientific	Hs01032433_m1	Waltham, MA, USA
CDKN1B (p27) TaqMan® Gene Expression Assay	Thermo Fisher Scientific	Hs00153277_m1	Waltham, MA, USA
DAPI	Thermo Fisher Scientific	62248	Waltham, MA, USA
Rat-Ki67-antibody, FITC	Thermo Fisher Scientific	11-5698-82	Waltham, MA, USA
Mouse-KRT17 antibody	Thermo Fisher Scientific	MA1-06325	Waltham, MA, USA
Rabbit-KRT17 antibody	Thermo Fisher Scientific	PA5-27949	Waltham, MA, USA
Mouse-KRT5 antibody	Thermo Fisher Scientific	MA5-12596	Waltham, MA, USA
Mouse-FN1 antibody	Thermo Fisher Scientific	MA5-11981	Waltham, MA, USA
Rat-SCGB1A1 antibody	RnD System	394324	Abingdon, UK
Alexa 488 donkey anti-rat	Thermo Fisher Scientific	21208	Waltham, MA, USA
Alexa 647 Goat anti-mouse	Thermo Fisher Scientific	A12235	Waltham, MA, USA
Alexa 488 Donkey anti-mouse	Thermo Fisher Scientific	A21202	Waltham, MA, USA

1. Adams TS, Schupp JC, Poli S, Ayaub EA, Neumark N, Ahangari F, Chu SG, Raby BA, DeIuliis G, Januszyk M, Duan Q, Arnett HA, Siddiqui A, Washko GR, Homer R, Yan X, Rosas IO,

Kaminski N. Single-cell RNA-seq reveals ectopic and aberrant lung-resident cell populations in idiopathic pulmonary fibrosis. *Science Advances* 2020; 6: eaba1983.

2. Habermann AC, Gutierrez AJ, Bui LT, Yahn SL, Winters NI, Calvi CL, Peter L, Chung M-I, Taylor CJ, Jetter C, Raju L, Roberson J, Ding G, Wood L, Sucre JMS, Richmond BW, Serezani AP, McDonnell WJ, Mallal SB, Bacchetta MJ, Loyd JE, Shaver CM, Ware LB, Bremner R, Walia R, Blackwell TS, Banovich NE, Kropski JA. Single-cell RNA sequencing reveals profibrotic roles of distinct epithelial and mesenchymal lineages in pulmonary fibrosis. *Science Advances* 2020; 6: eaba1972.
3. Reyfman PA, Walter JM, Joshi N, Anekalla KR, McQuattie-Pimentel AC, Chiu S, Fernandez R, Akbarpour M, Chen CI, Ren Z, Verma R, Abdala-Valencia H, Nam K, Chi M, Han S, Gonzalez-Gonzalez FJ, Soberanes S, Watanabe S, Williams KJN, Flozak AS, Nicholson TT, Morgan VK, Winter DR, Hinchcliff M, Hrusch CL, Guzy RD, Bonham CA, Sperling AI, Bag R, Hamanaka RB, Mutlu GM, Yeldandi AV, Marshall SA, Shilatifard A, Amaral LAN, Perlman H, Sznajder JI, Argento AC, Gillespie CT, Dematte J, Jain M, Singer BD, Ridge KM, Lam AP, Bharat A, Bhorade SM, Gottardi CJ, Budinger GRS, Misharin AV. Single-Cell Transcriptomic Analysis of Human Lung Provides Insights into the Pathobiology of Pulmonary Fibrosis. *American journal of respiratory and critical care medicine* 2019; 199: 1517-1536.
4. Travaglini KJ, Nabhan AN, Penland L, Sinha R, Gillich A, Sit RV, Chang S, Conley SD, Mori Y, Seita J, Berry GJ, Shrager JB, Metzger RJ, Kuo CS, Neff N, Weissman IL, Quake SR, Krasnow MA. A molecular cell atlas of the human lung from single-cell RNA sequencing. *Nature* 2020; 587: 619-625.
5. Kathiriya JJ, Wang C, Brumwell A, Cassandras M, Le Saux C, Wolters P, Matthay M, Chapman HA, Peng T. Human alveolar Type 2 epithelium transdifferentiates into metaplastic KRT5+ basal cells during alveolar repair. *bioRxiv* 2020: 2020.2006.2006.136713.
6. Strunz M, Simon LM, Ansari M, Kathiriya JJ, Angelidis I, Mayr CH, Tsidiridis G, Lange M, Mattner LF, Yee M, Ogar P, Sengupta A, Kukhtevich I, Schneider R, Zhao Z, Voss C, Stoeger T, Neumann JHL, Hilgendorff A, Behr J, O'Reilly M, Lehmann M, Burgstaller G, Königshoff M, Chapman HA, Theis FJ, Schiller HB. Alveolar regeneration through a Krt8+ transitional stem cell state that persists in human lung fibrosis. *Nature Communications* 2020; 11: 3559.
