# Supplementary figures and images for: Alveolar Basal Cells Differentiate towards Secretory Epithelial- and Aberrant Basaloid-like Cells In Vitro
Source: Cells. 2022 Jun 2;11(11):1820. doi: 10.3390/cells11111820 (PMC9180703; doi:10.3390/cells11111820)

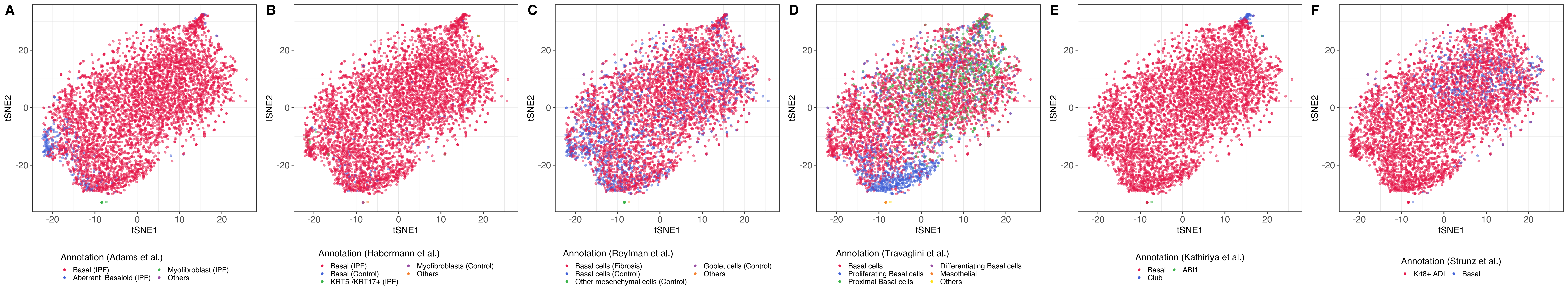

Supplement: Supplementary file 1 [file cells-11-01820-s001.zip › New supplement figure S7.pdf]

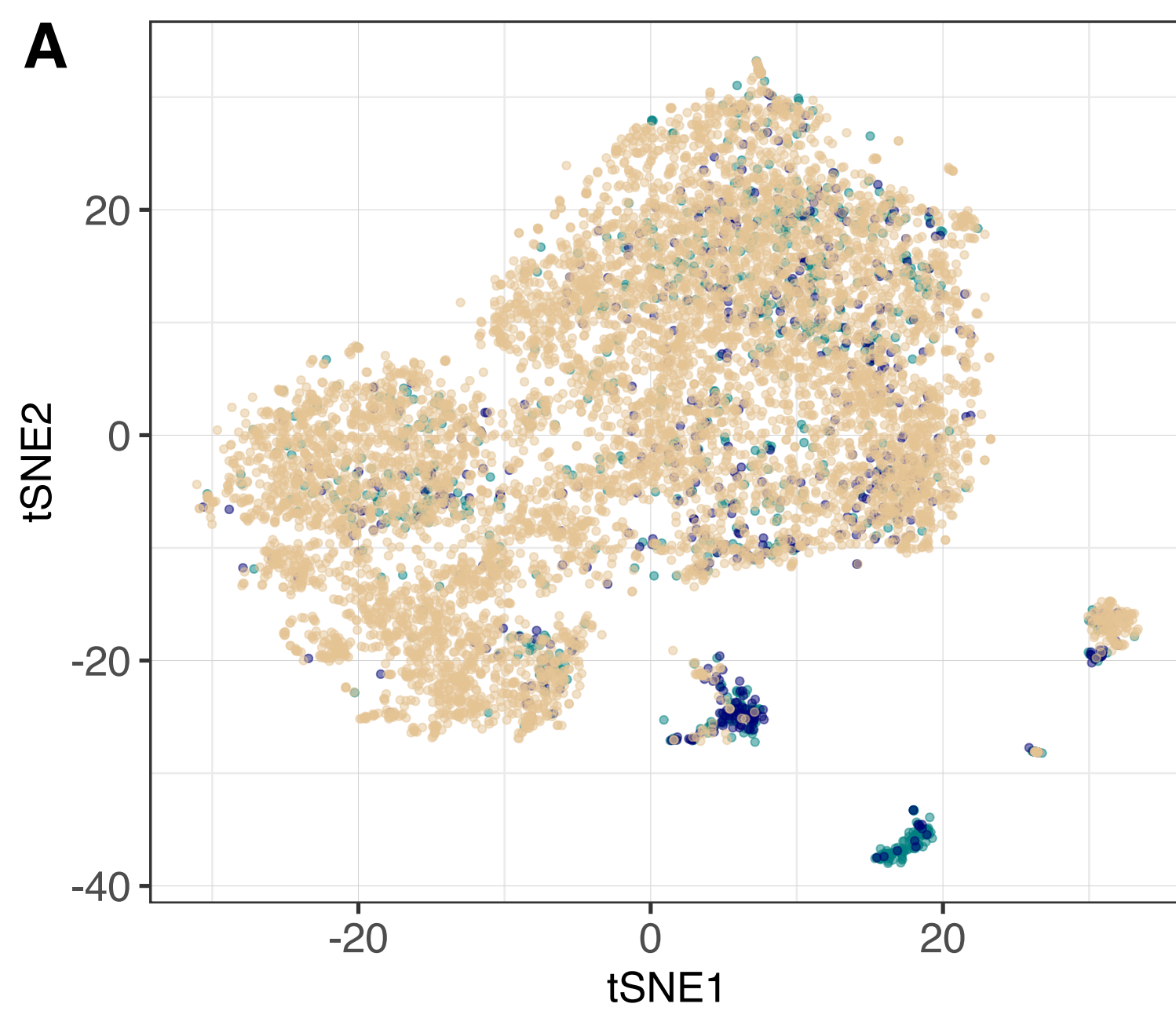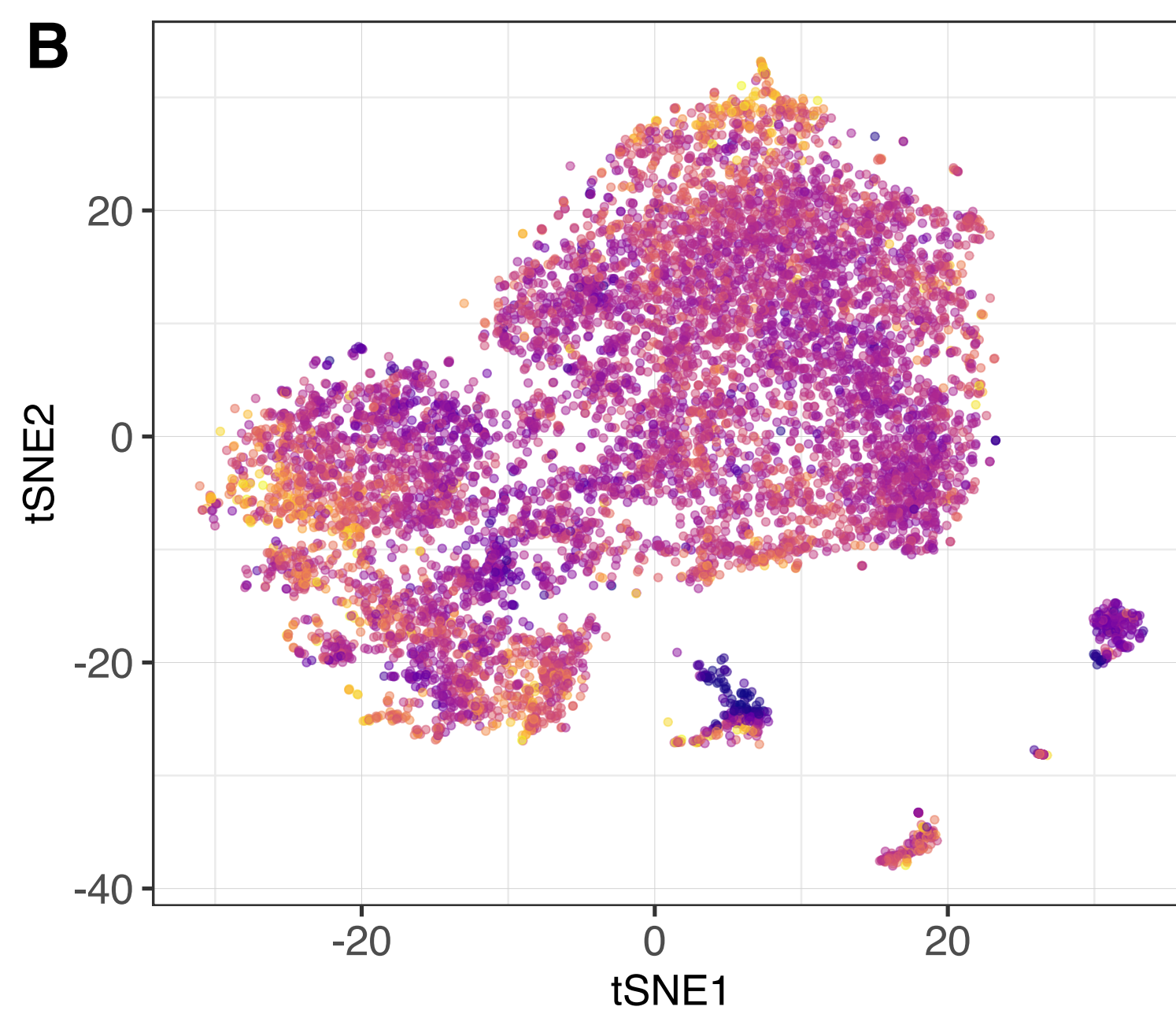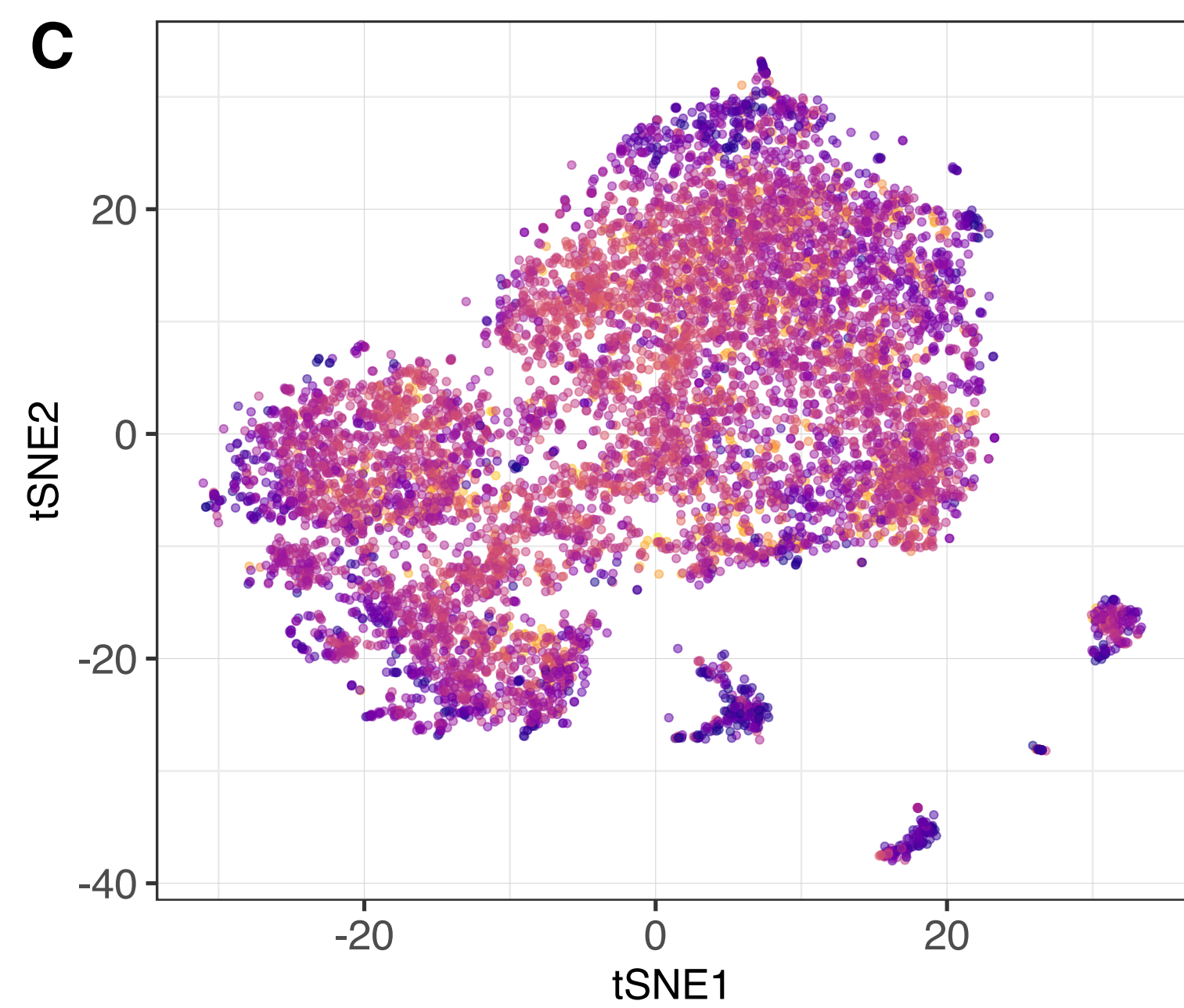

Supplement: Supplementary file 1 [file cells-11-01820-s001.zip › Supplement Figure S1.pdf]

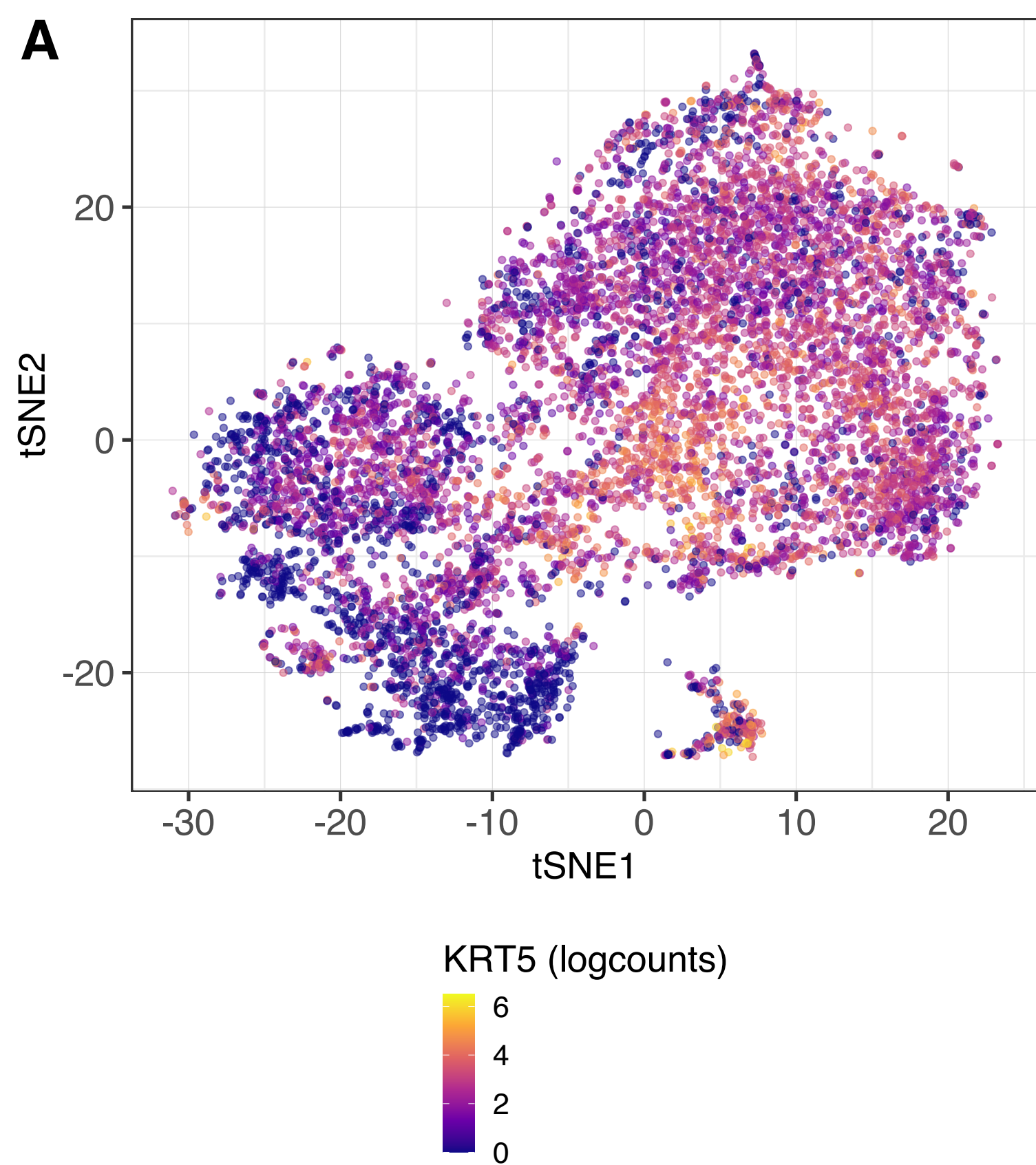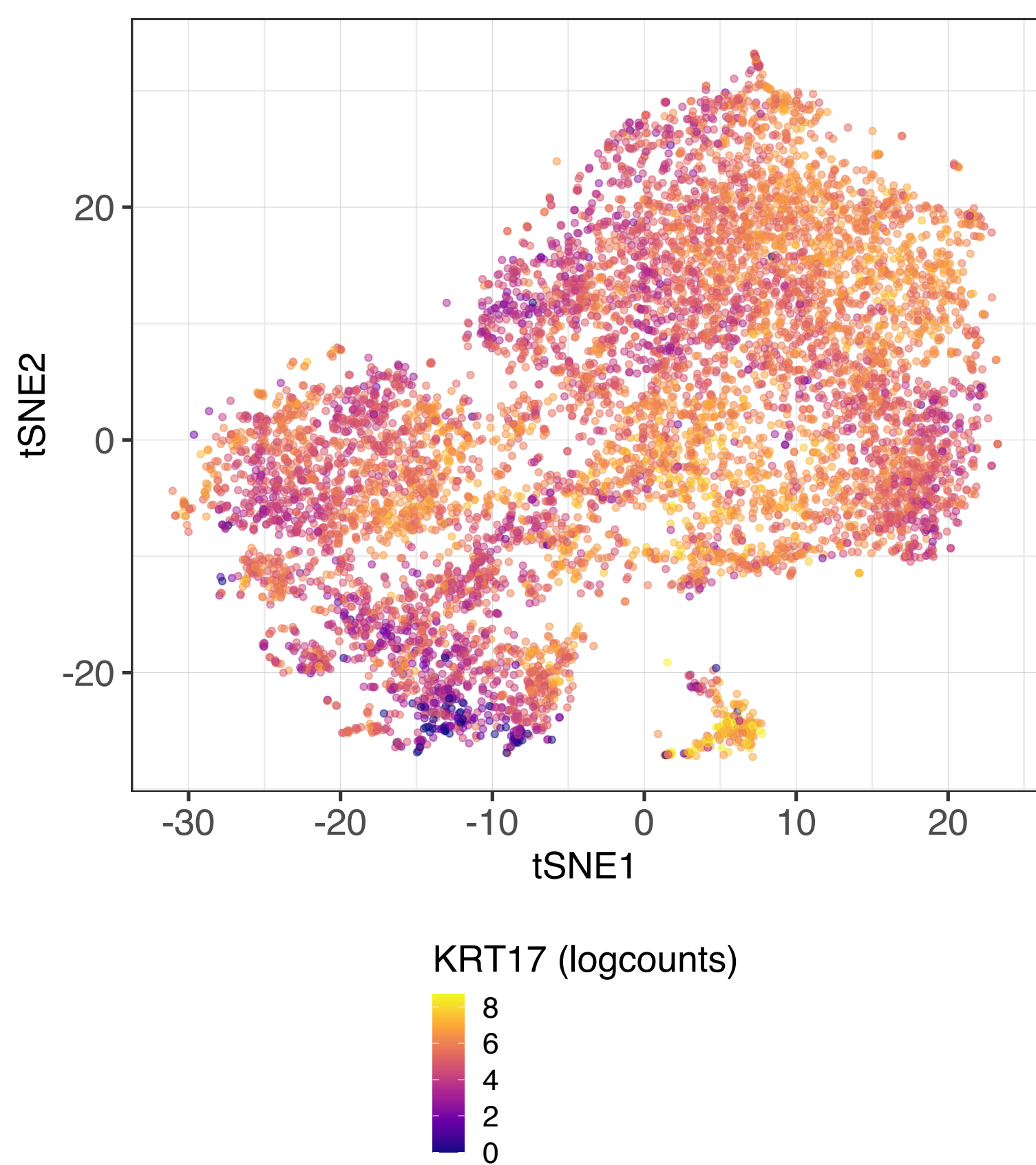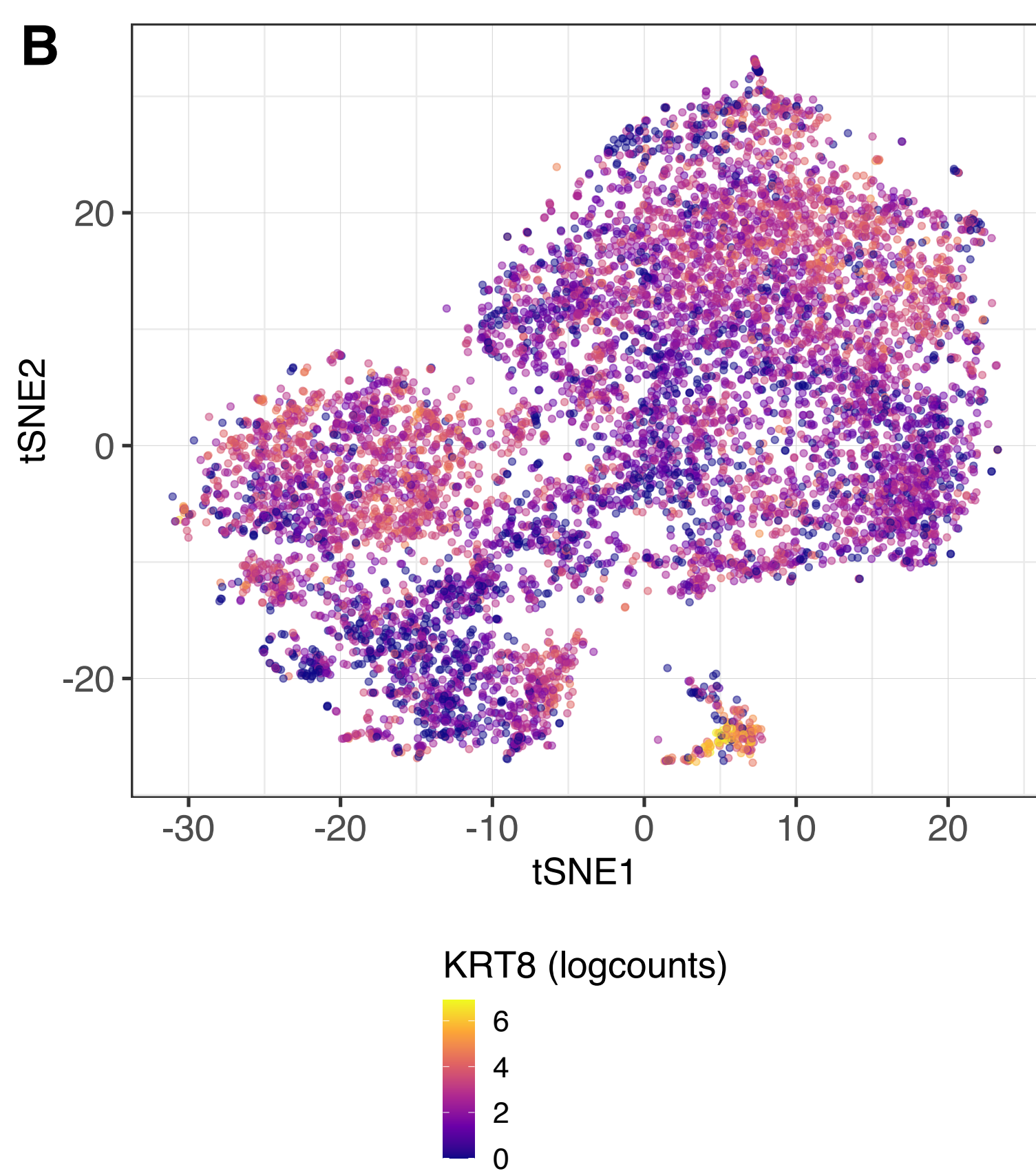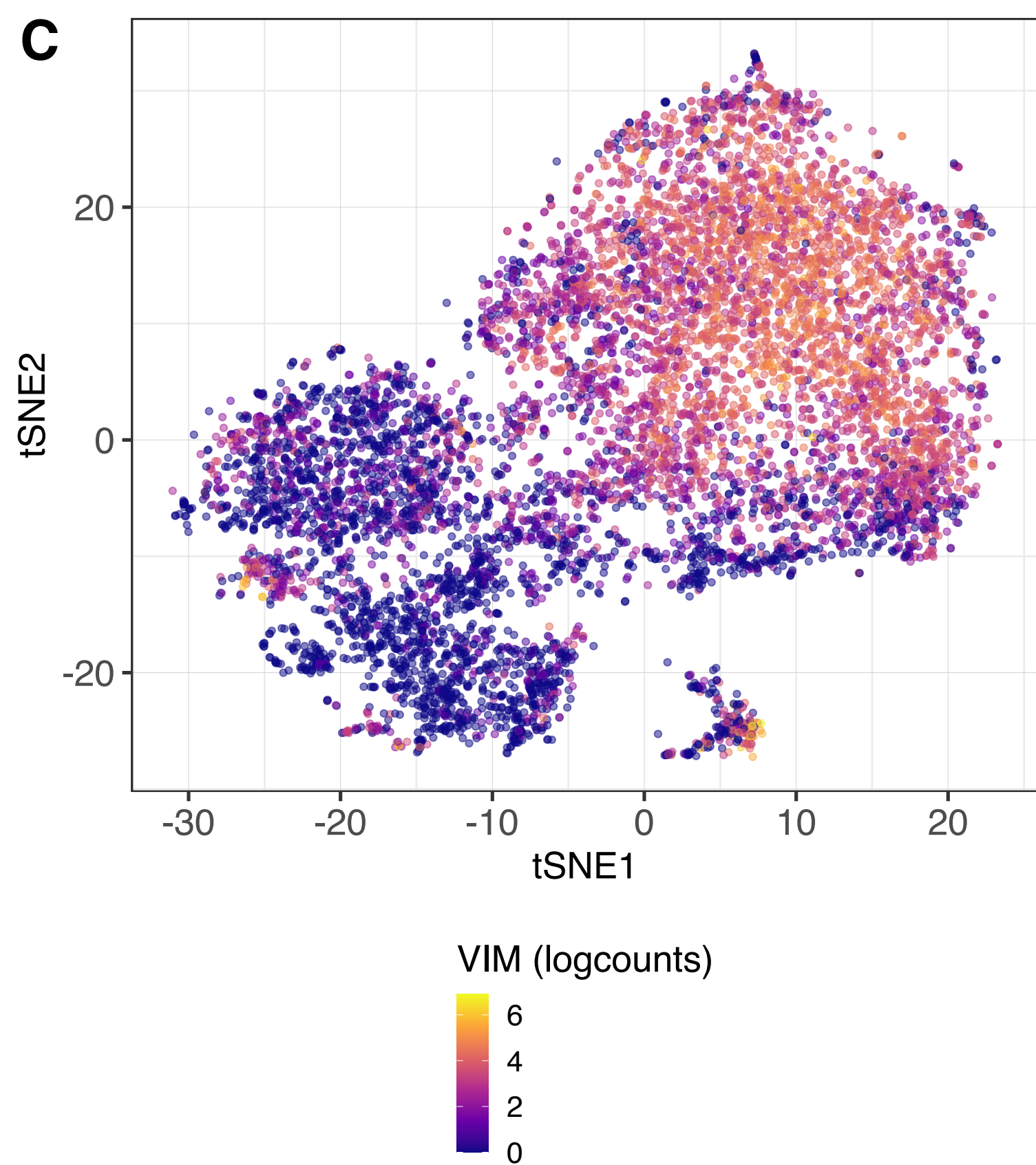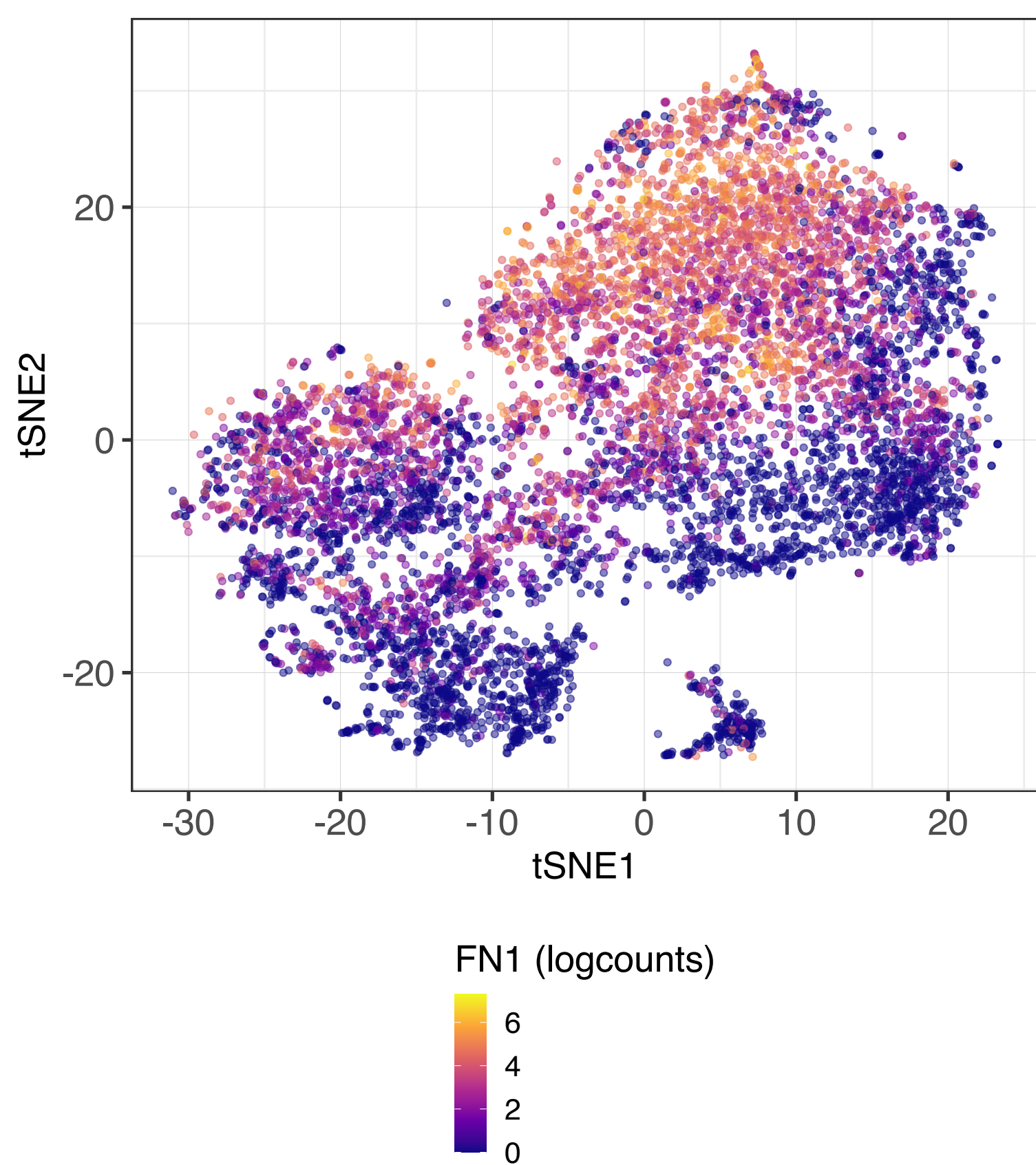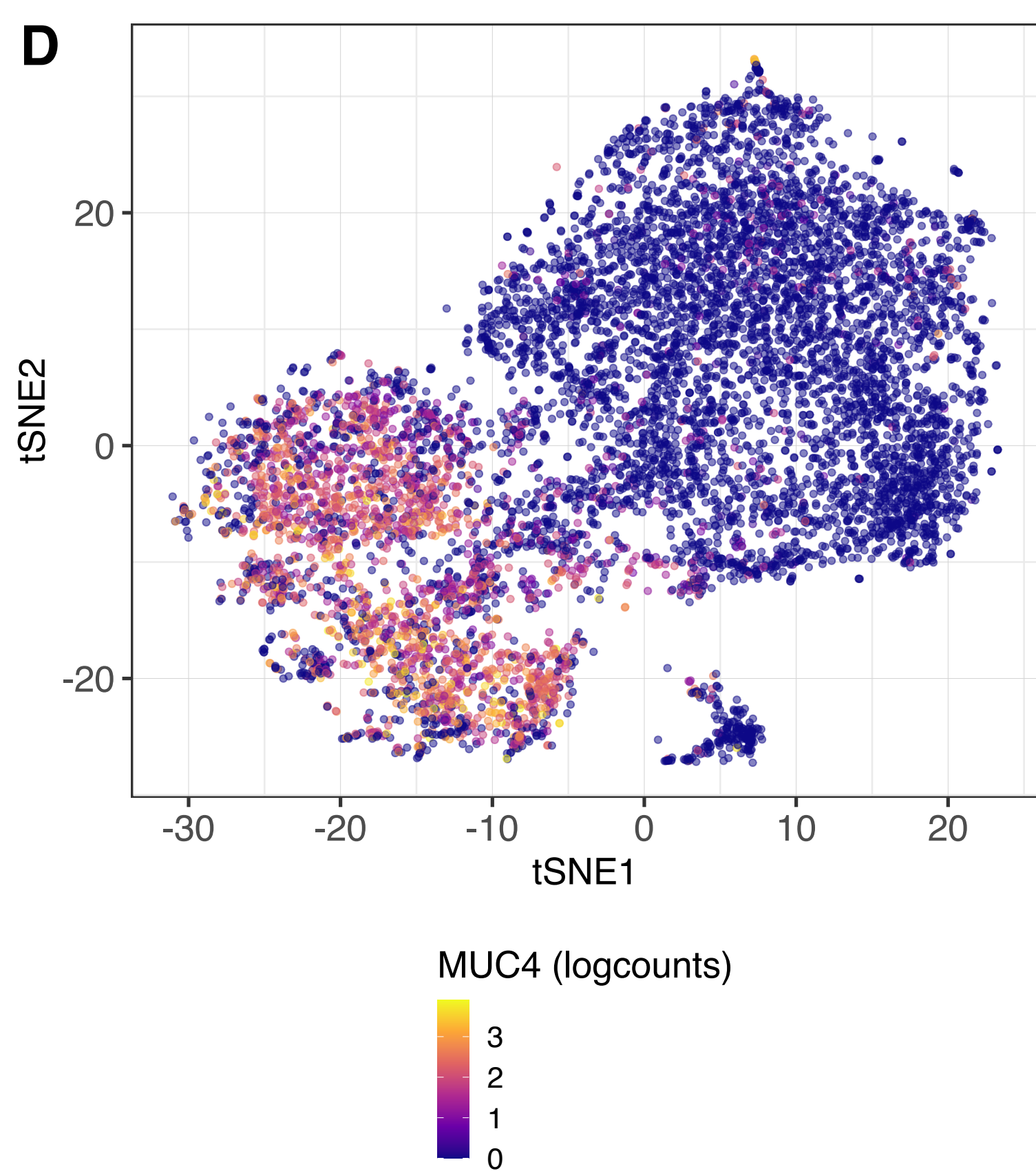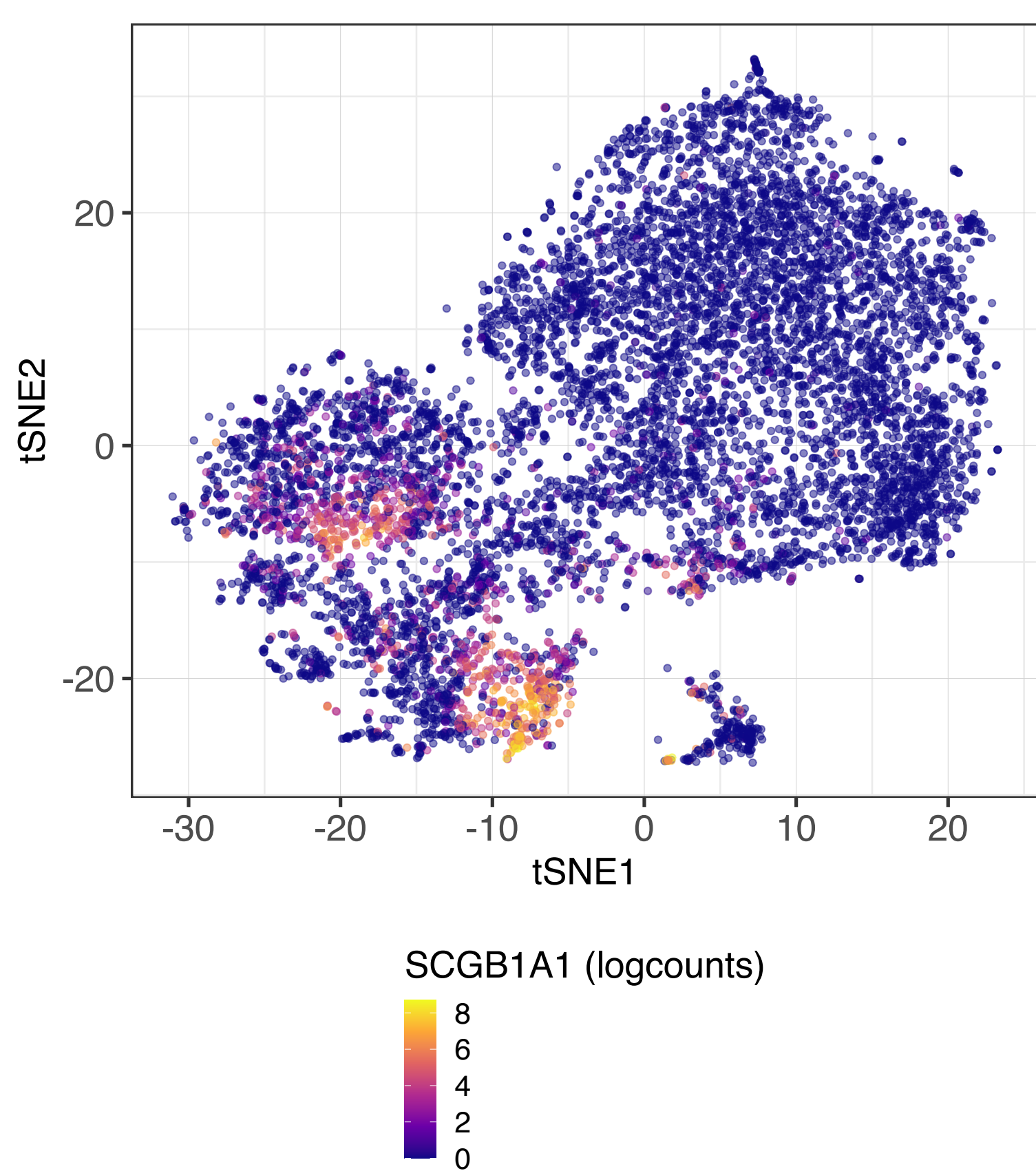

Supplement: Supplementary file 1 [file cells-11-01820-s001.zip › Supplement Figure S3.pdf]

**A**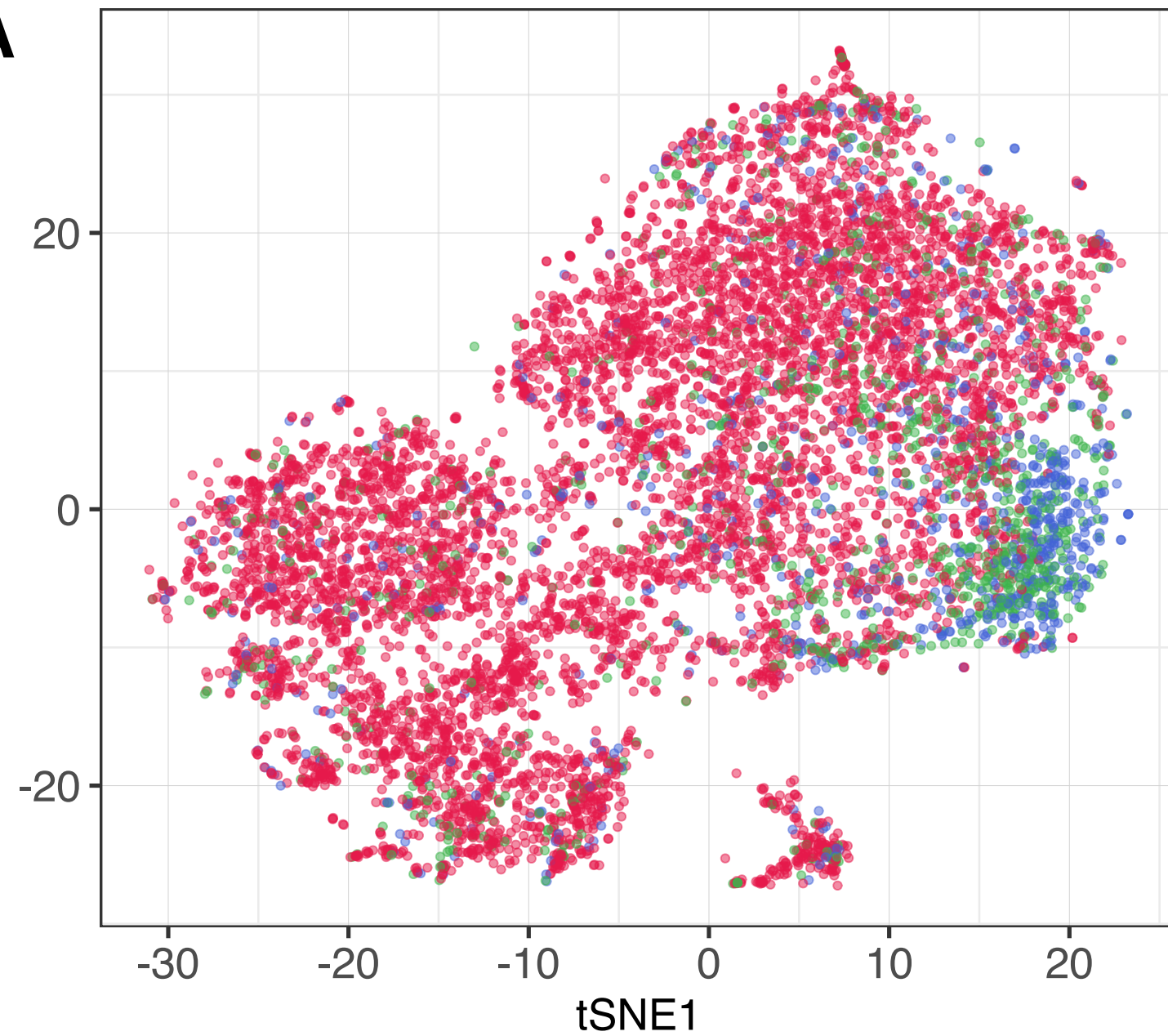

Cell-cycle phase

- G1
- G2M
- S

**B**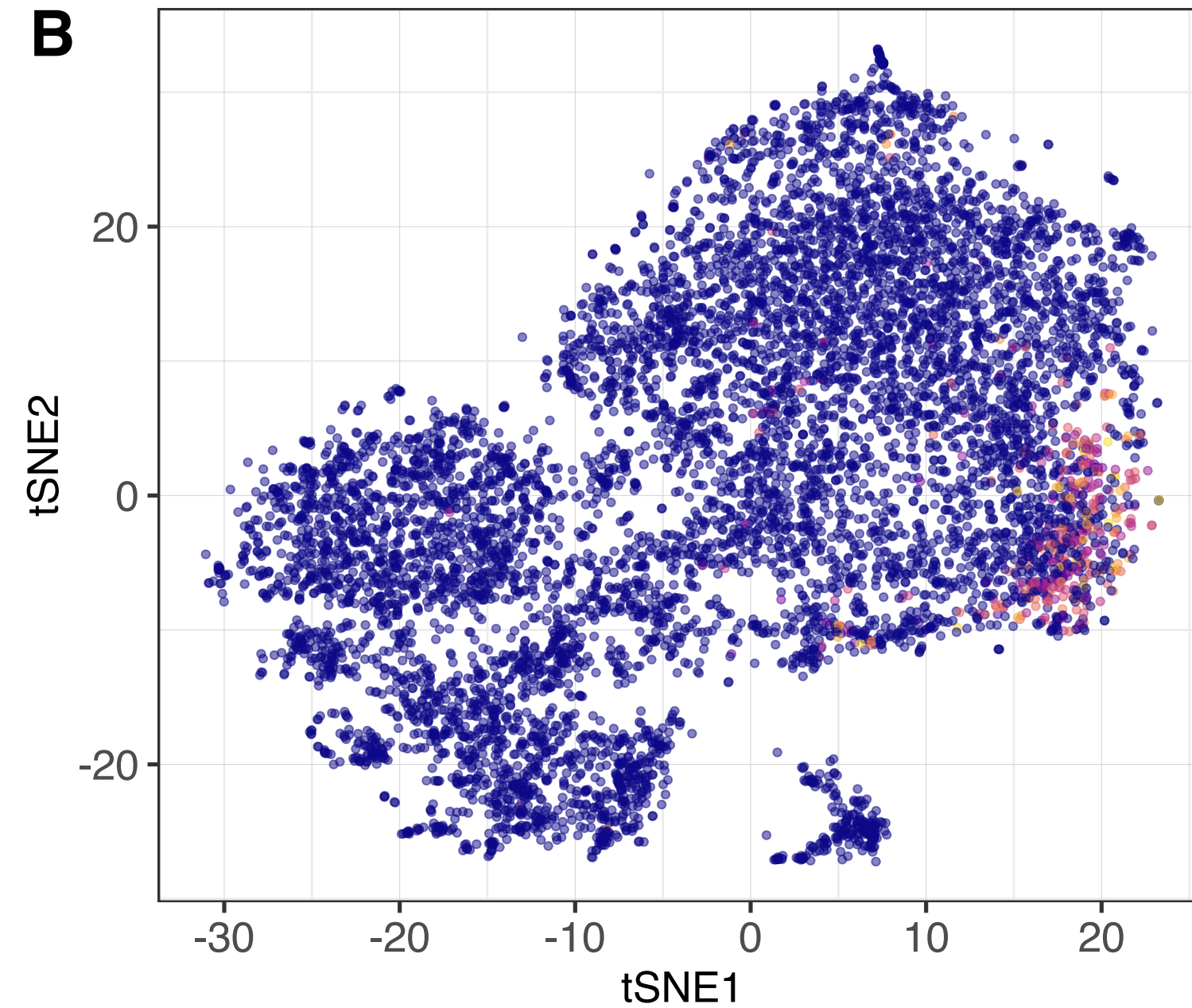

MKI67 (logcounts)

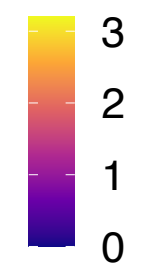**C**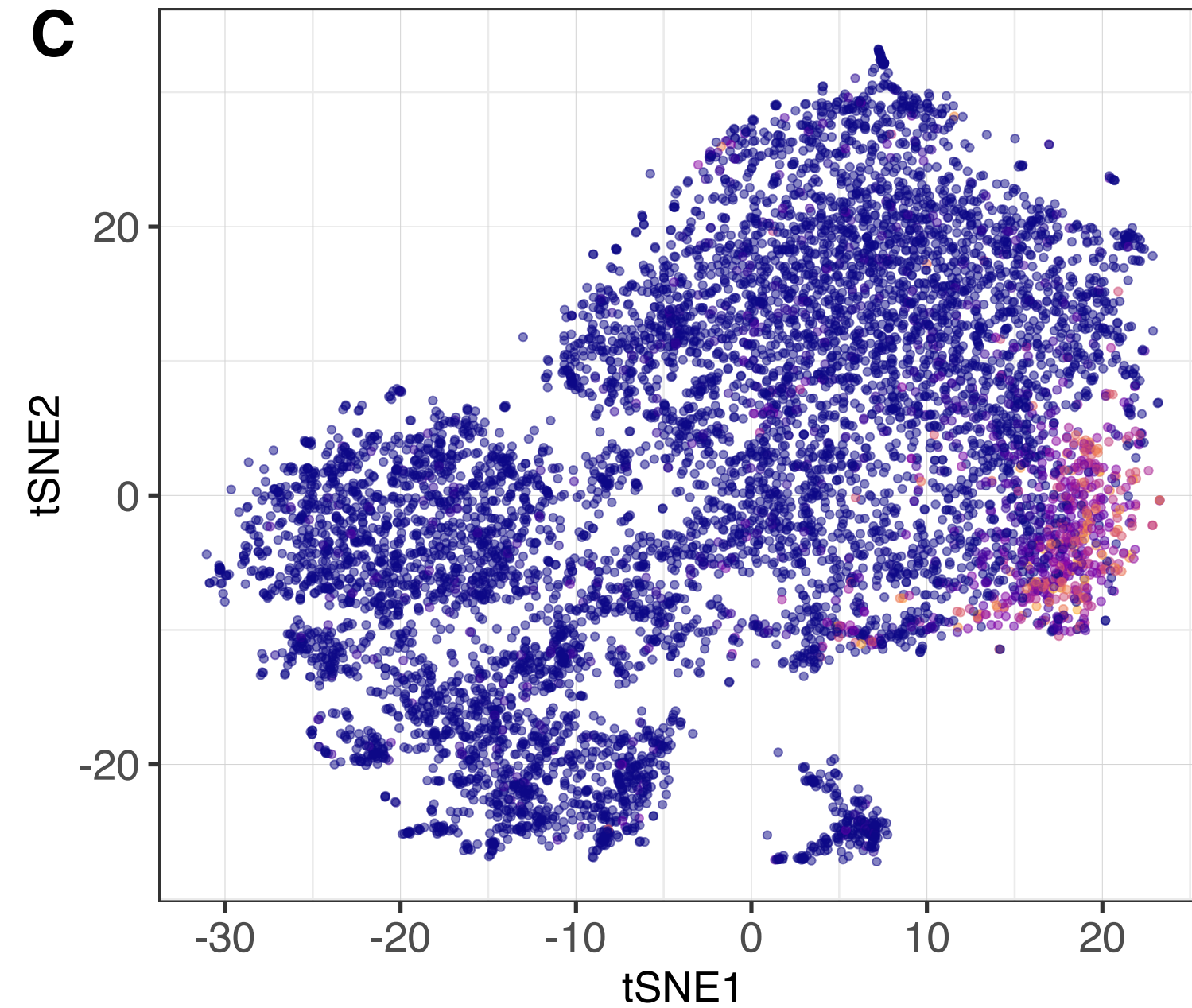

Proliferative signature (Travaglini et al.)

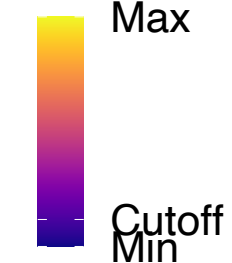

Supplement: Supplementary file 1 [file cells-11-01820-s001.zip › Supplement Figure S4.pdf]

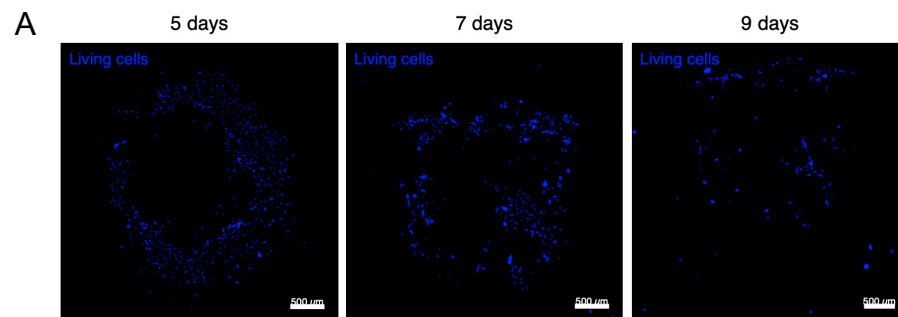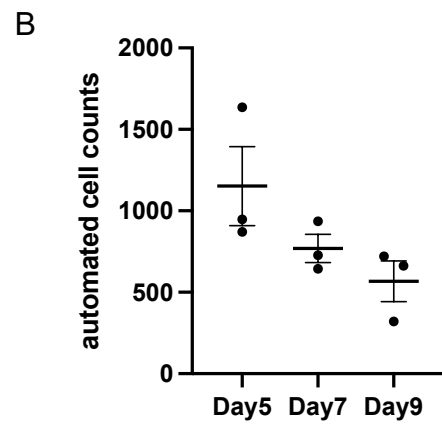

Supplement: Supplementary file 1 [file cells-11-01820-s001.zip › Supplement Figure S5.pdf]

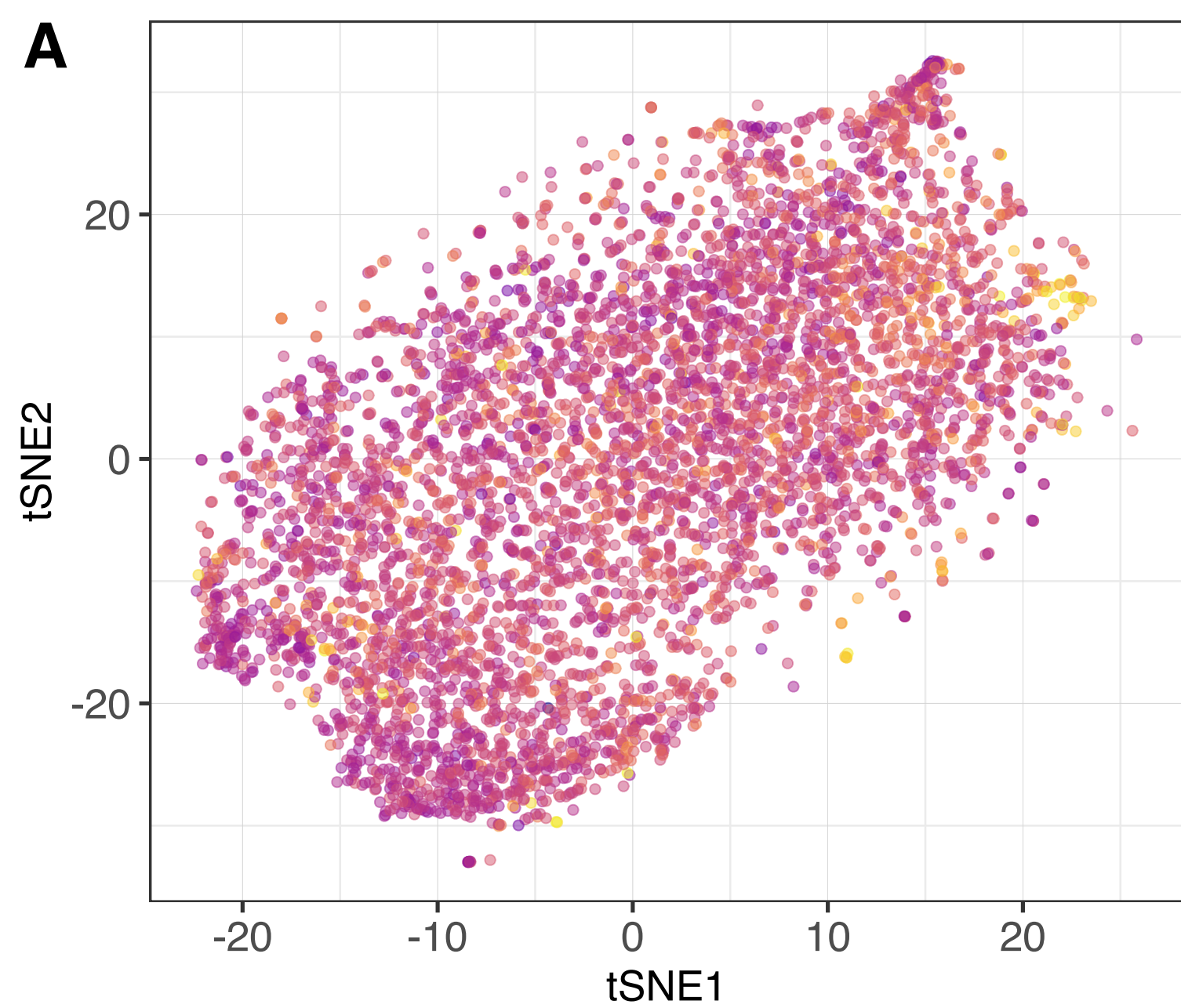

%MT reads

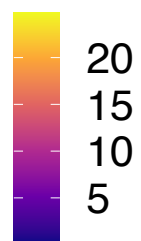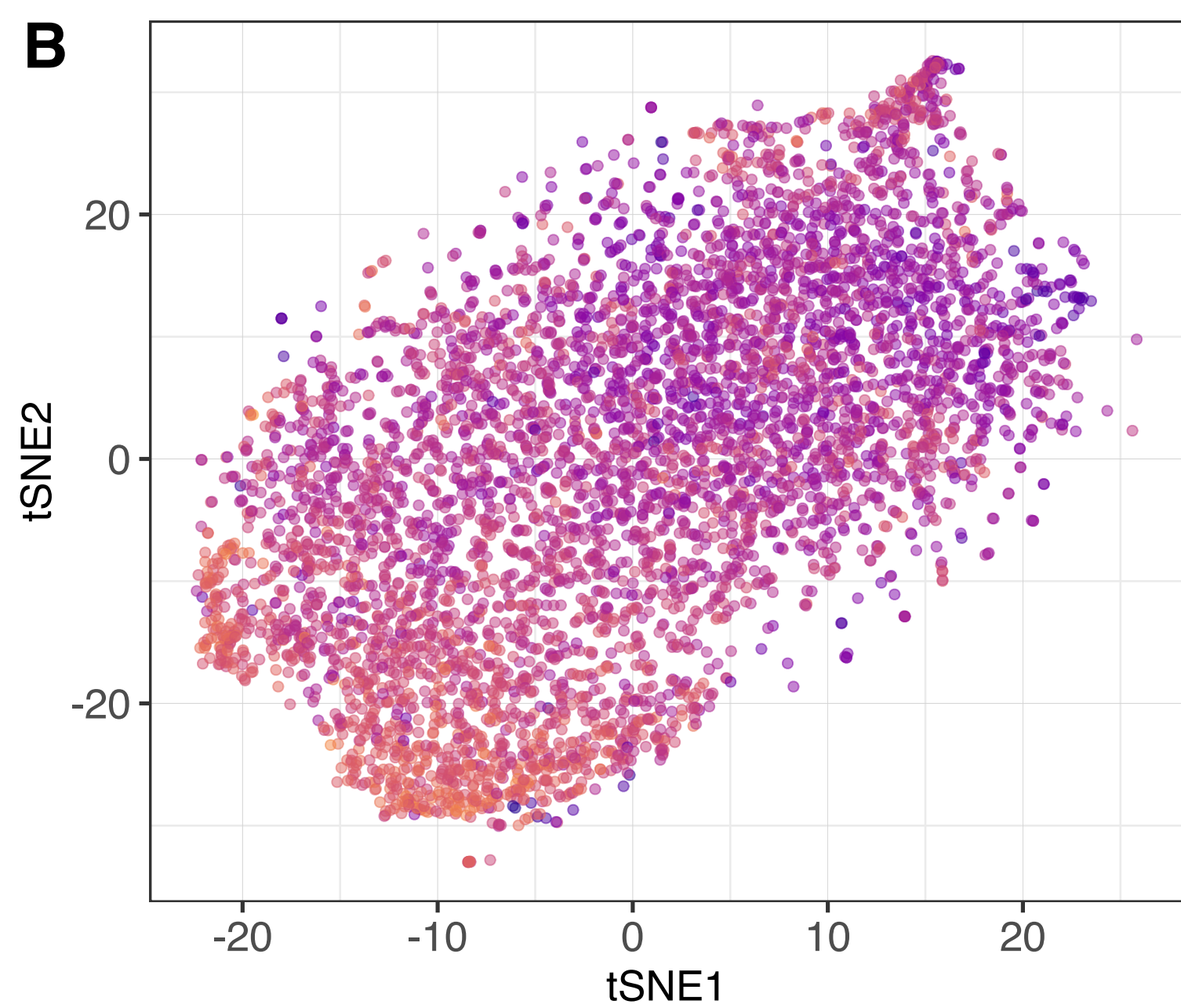

Number of detected genes

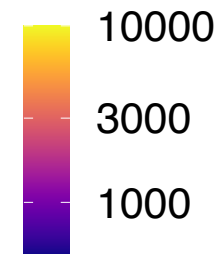

Supplement: Supplementary file 1 [file cells-11-01820-s001.zip › Supplement Figure S6.pdf]

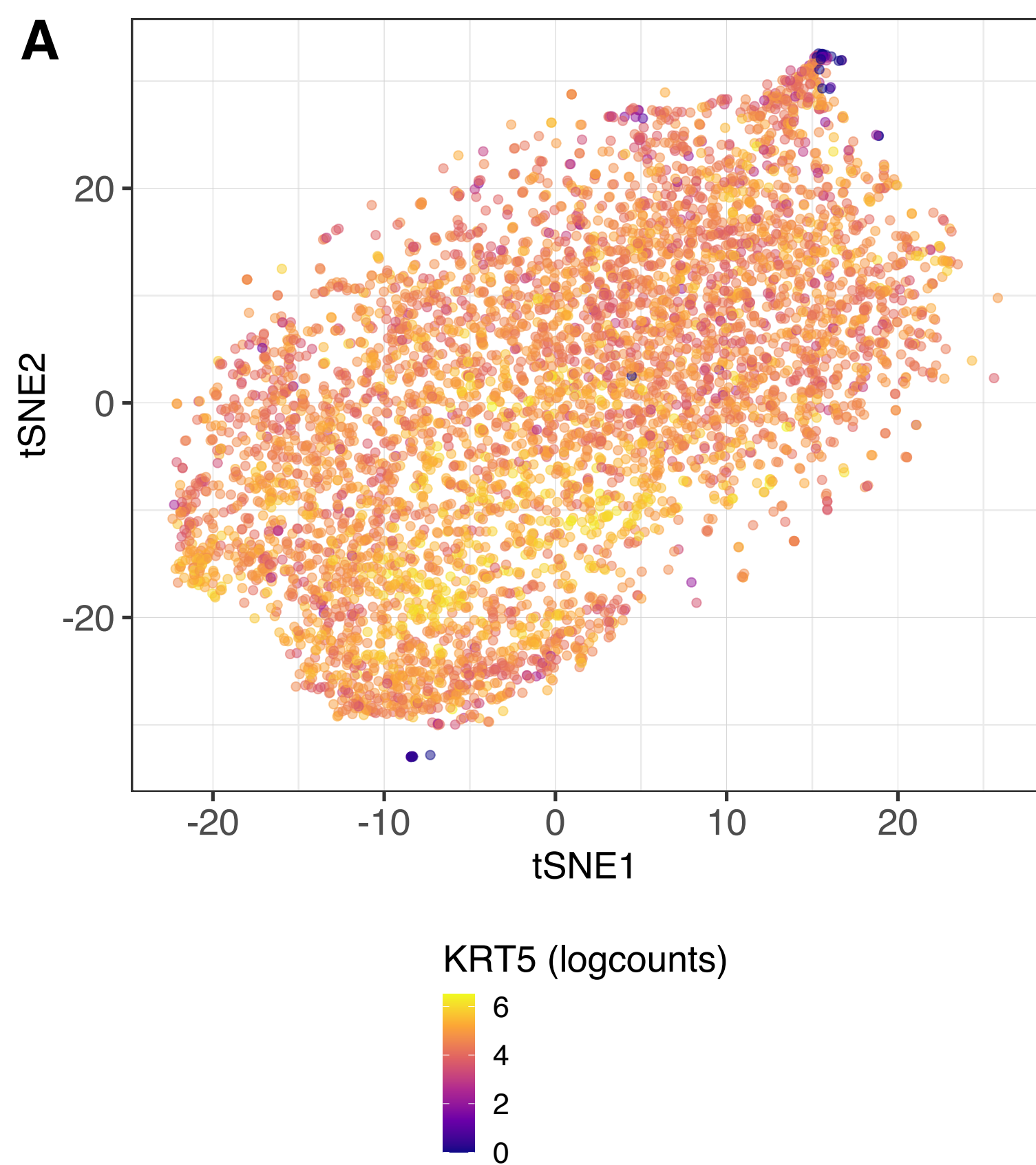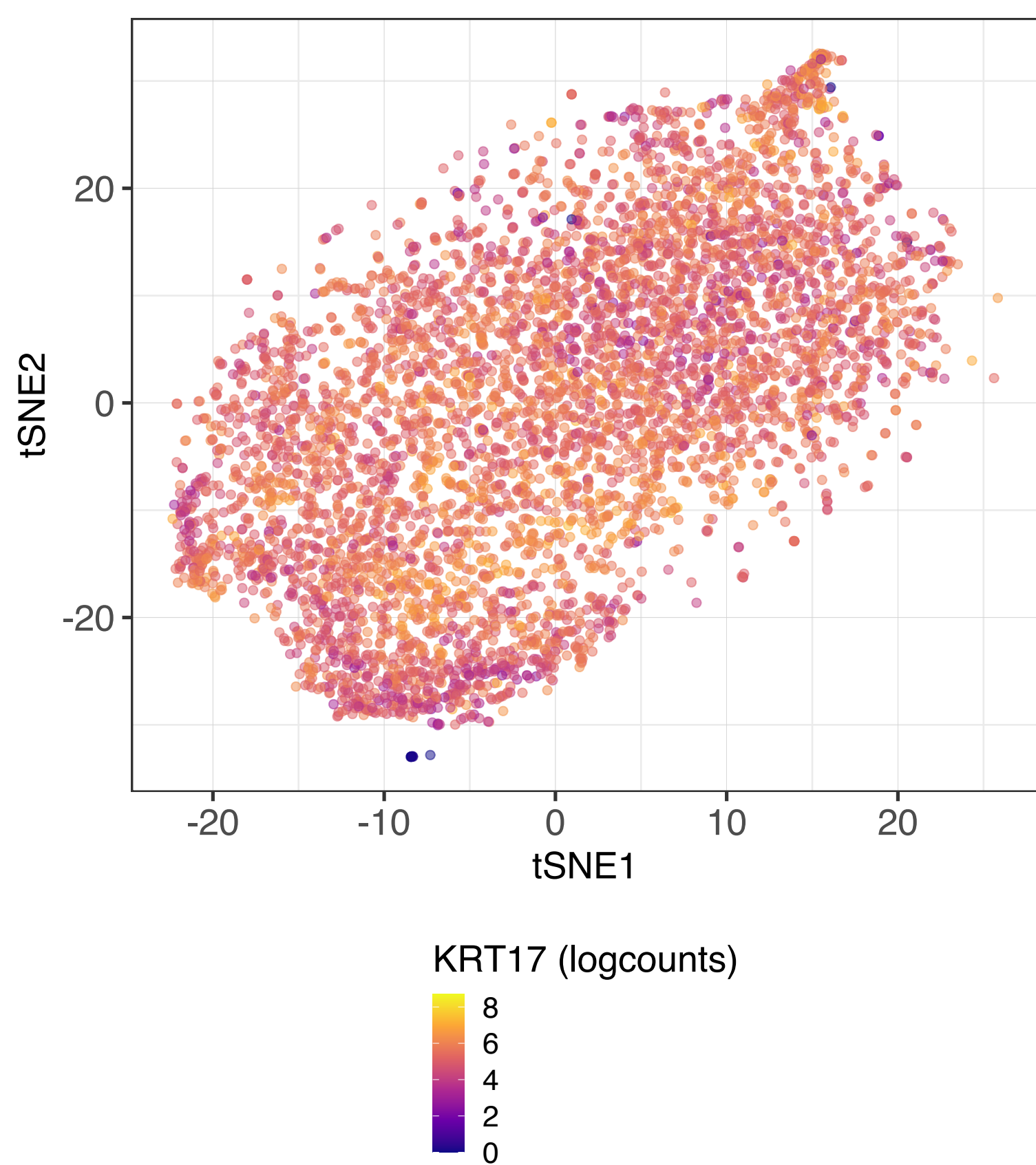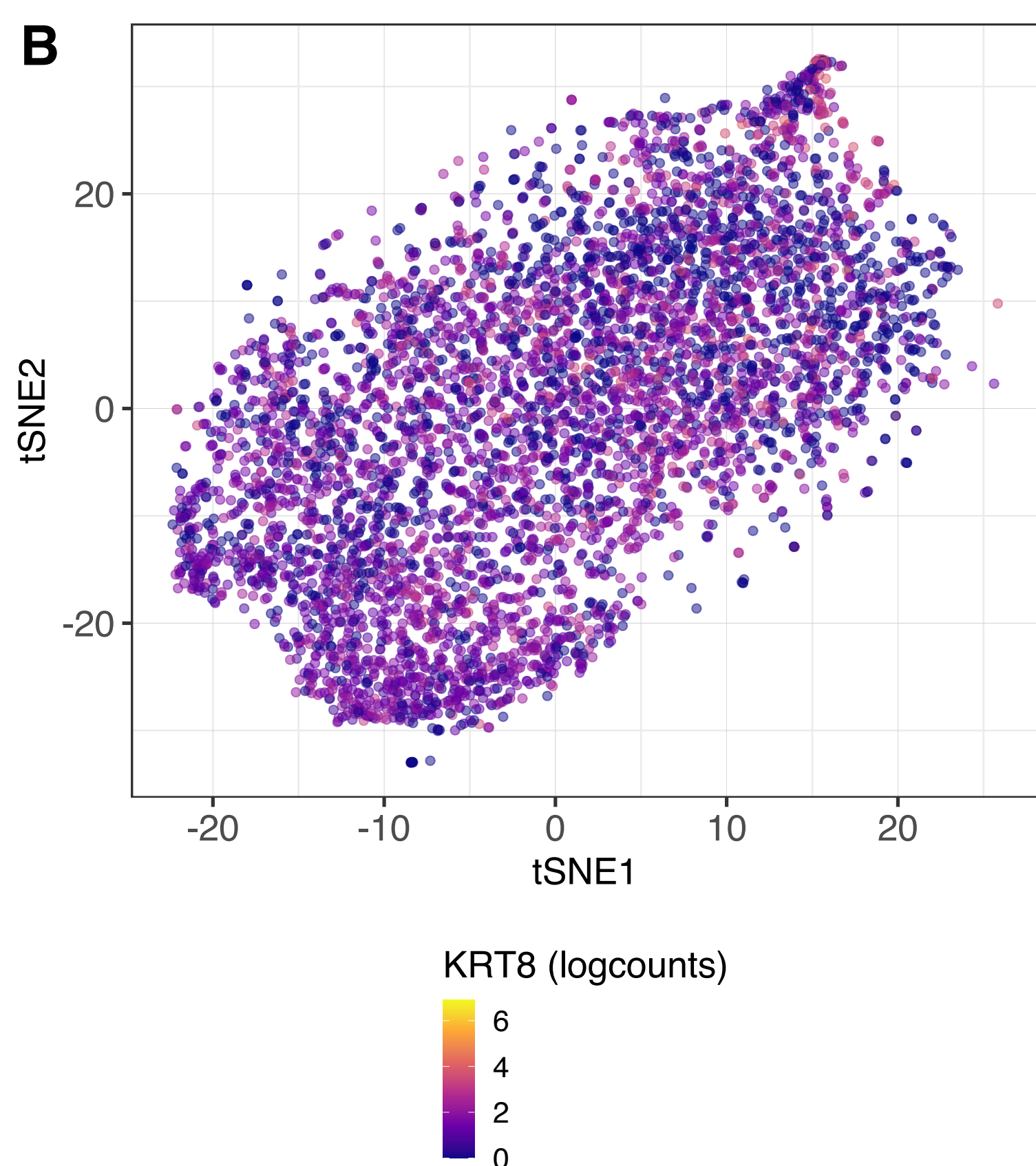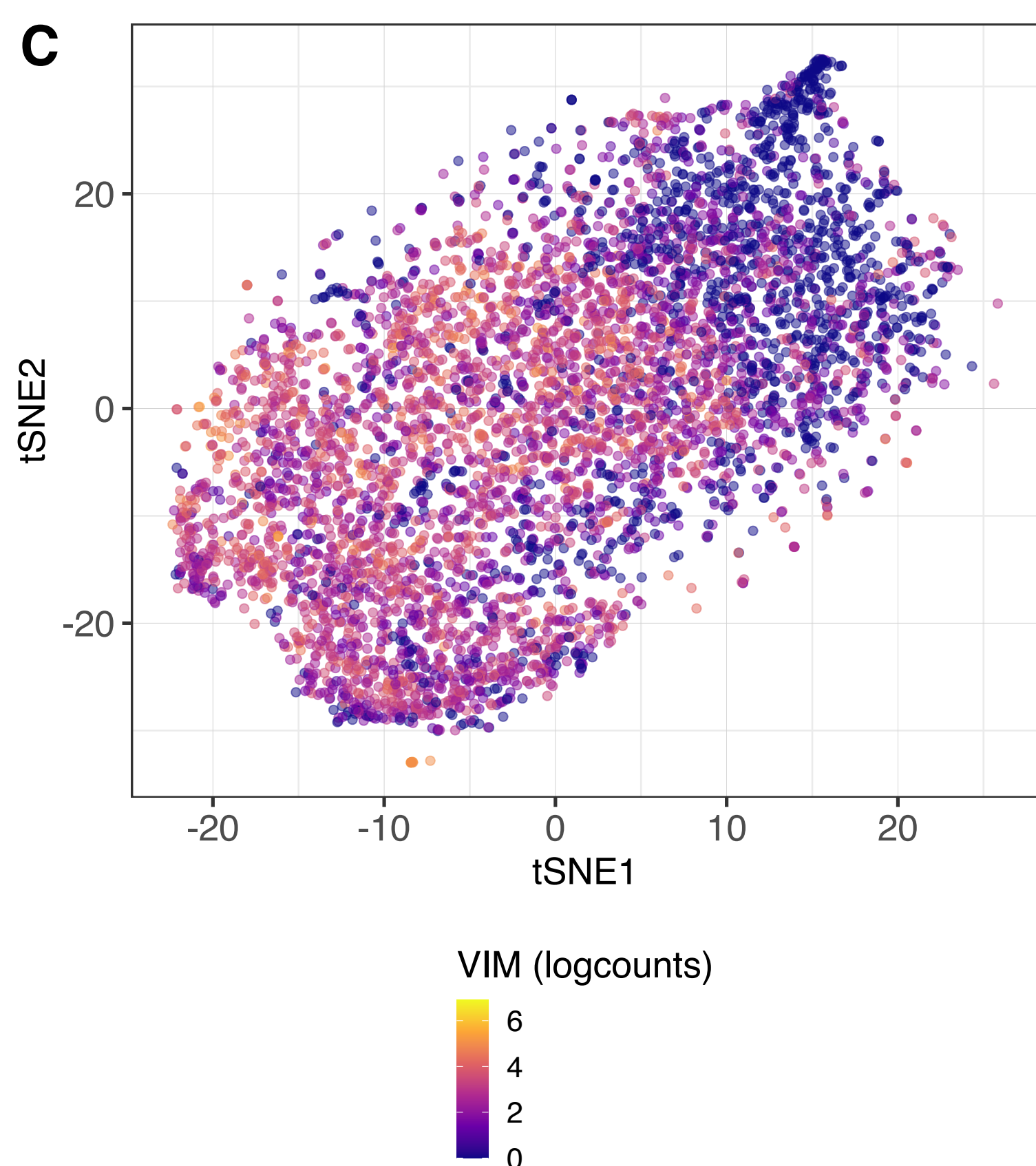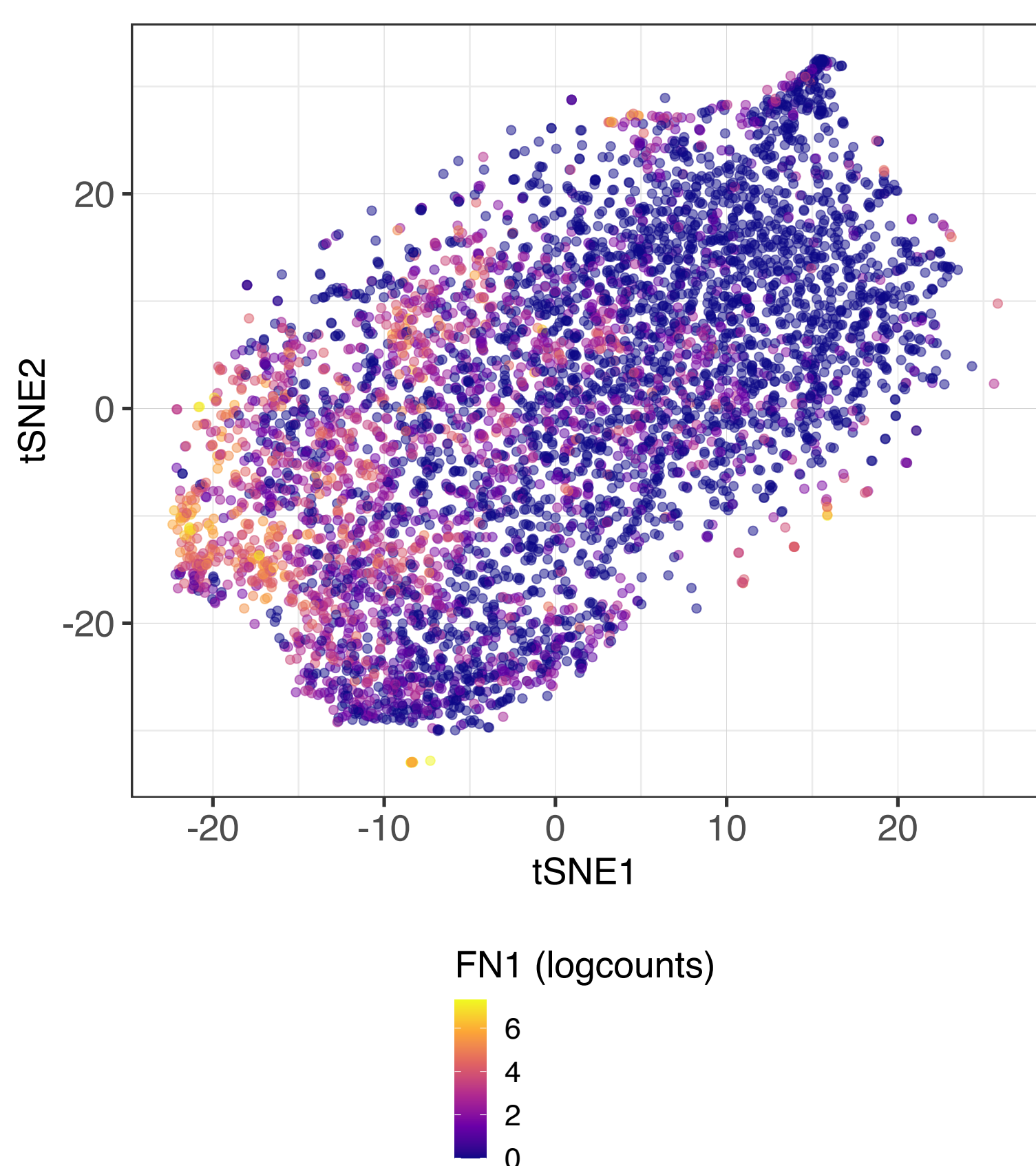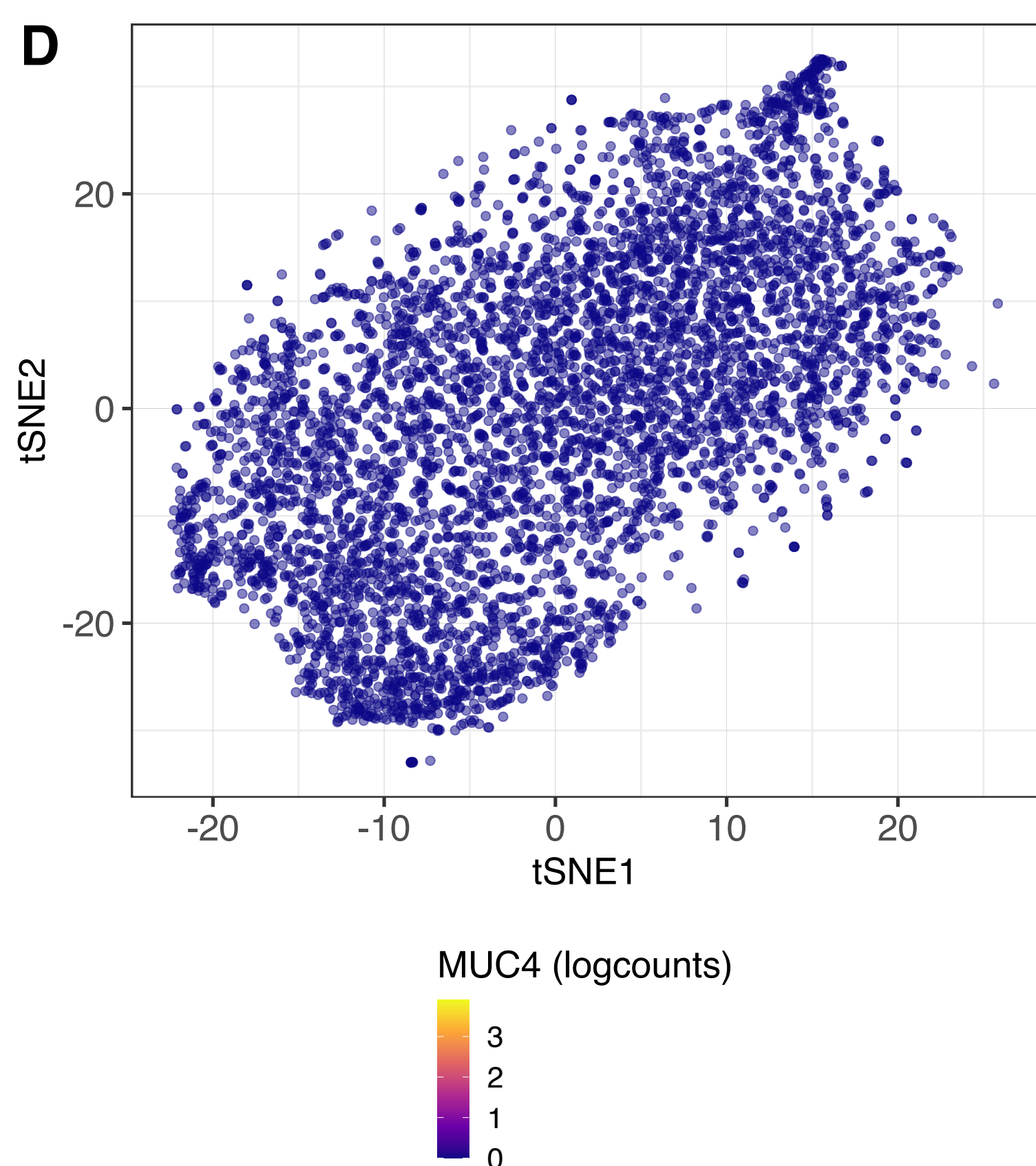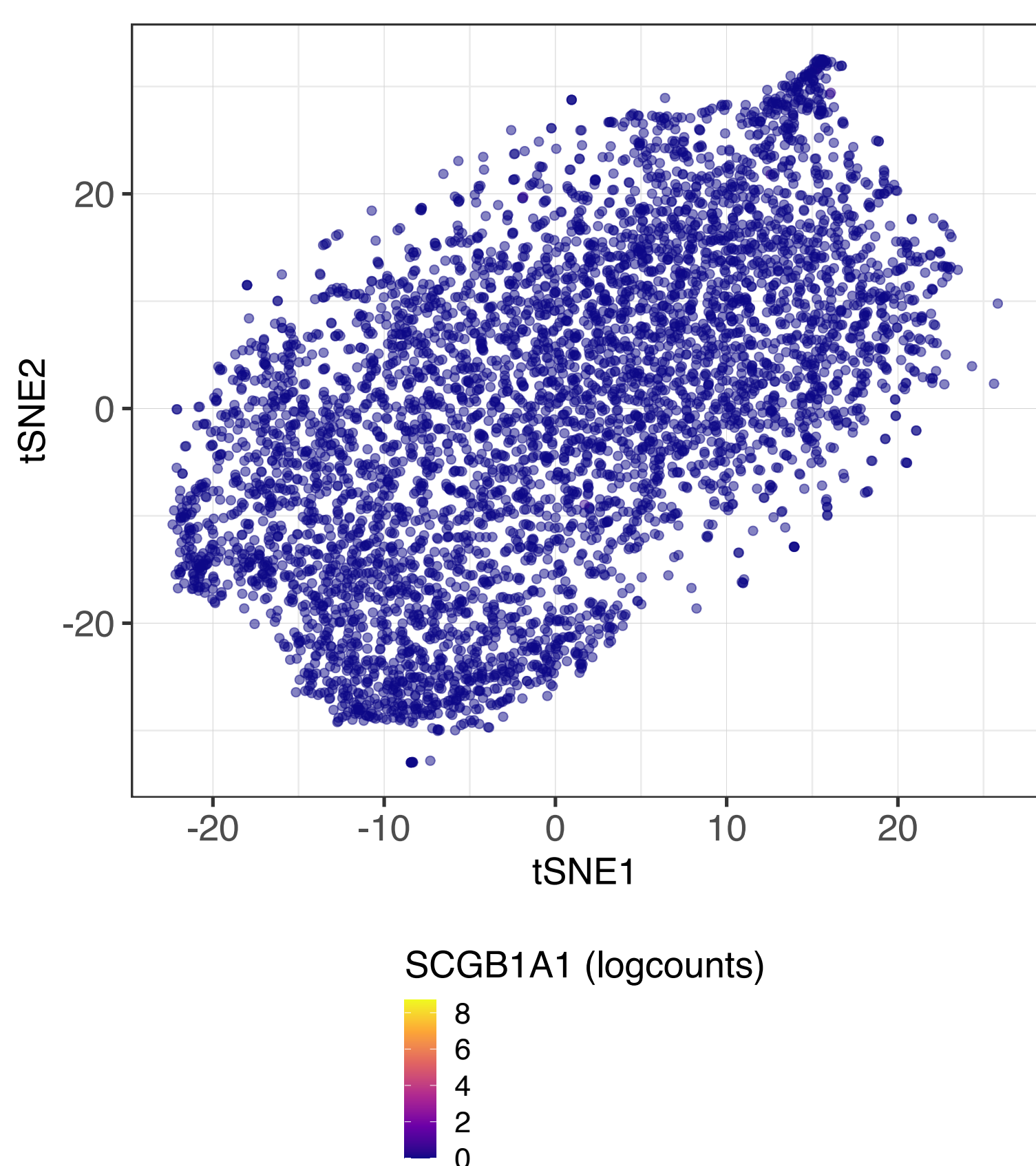

Supplement: Supplementary file 1 [file cells-11-01820-s001.zip › Supplement Figure S8.pdf]

**A**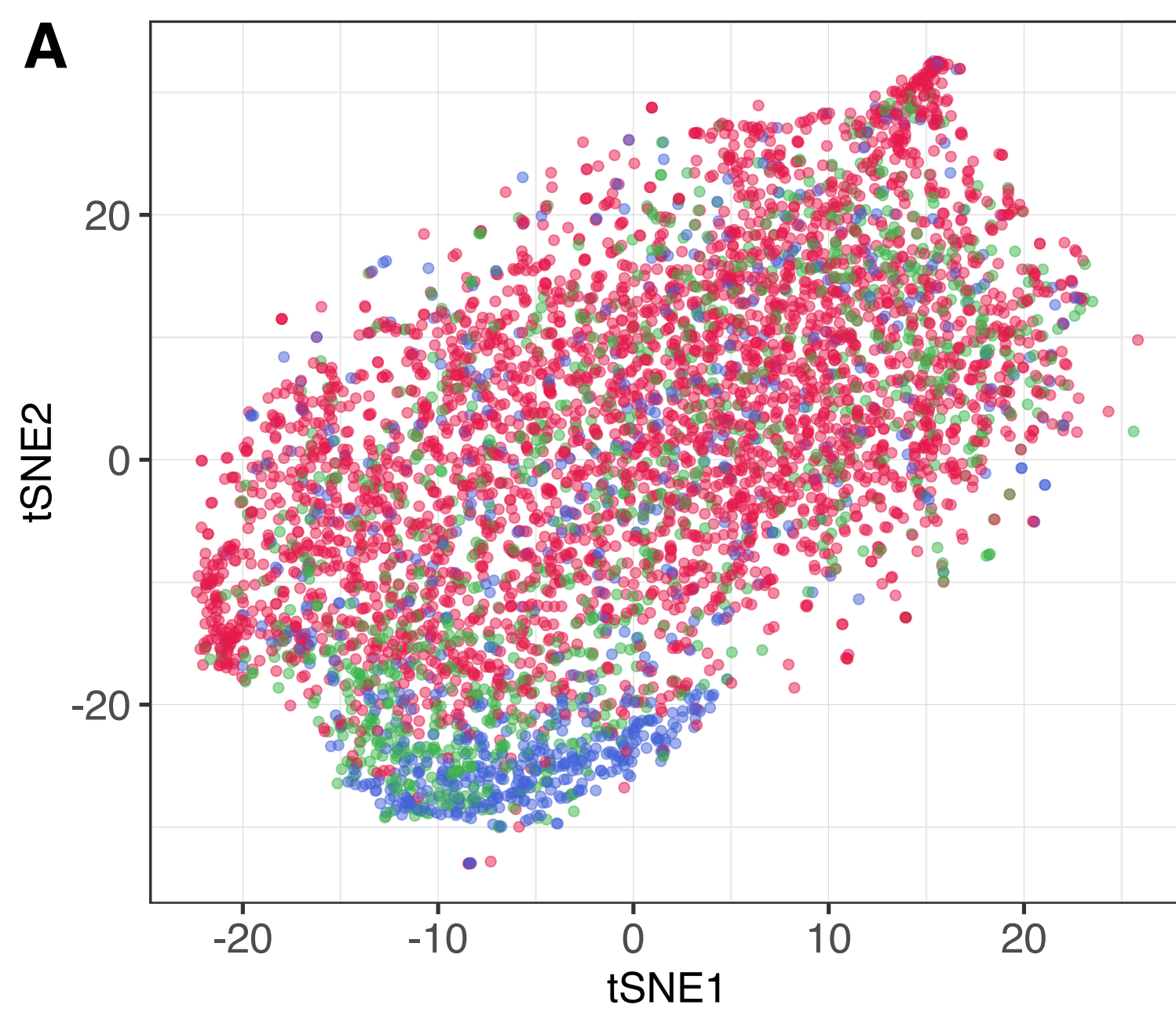

Cell-cycle phase

- G1
- G2M
- S

**B**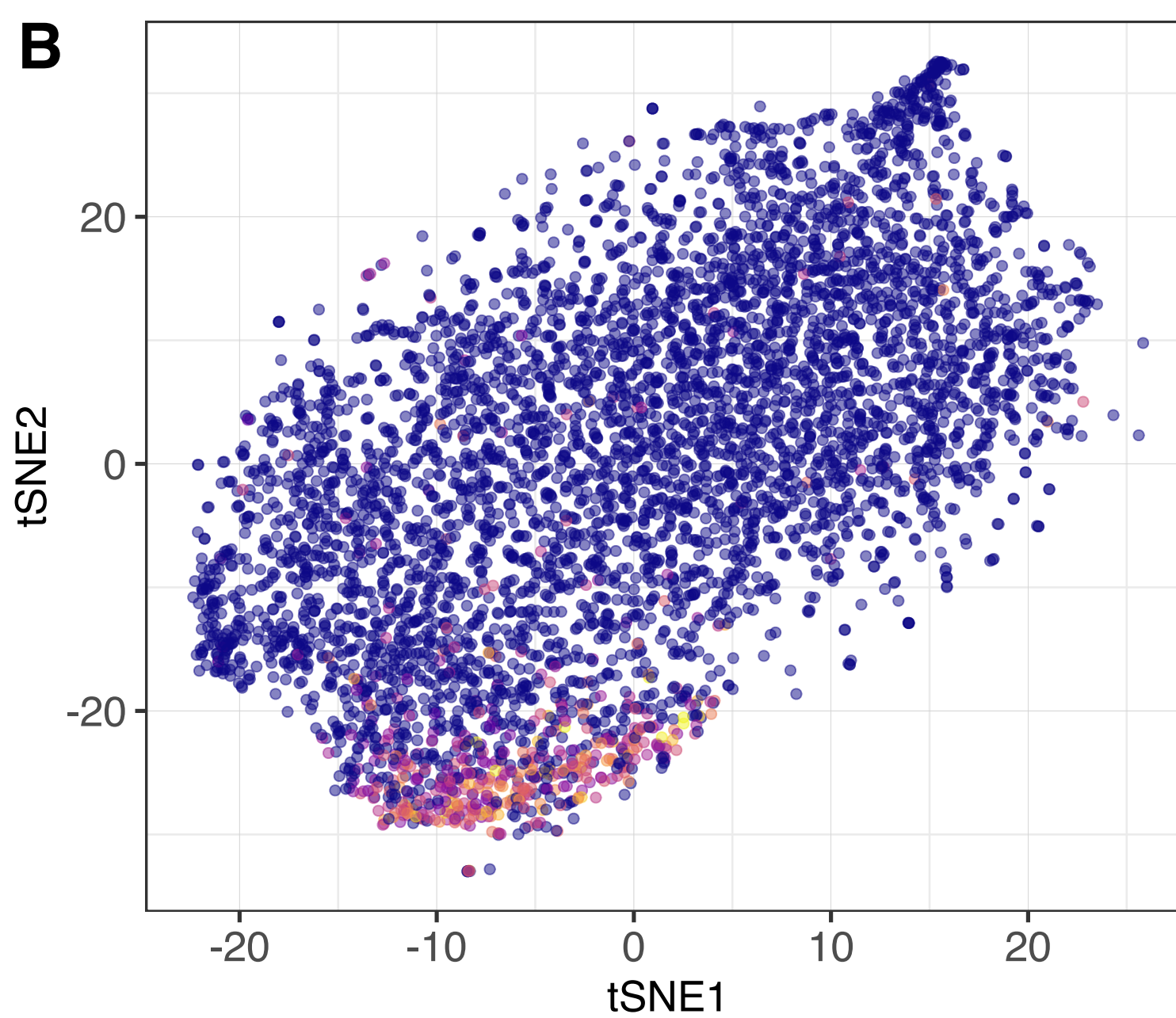

MKI67 (logcounts)

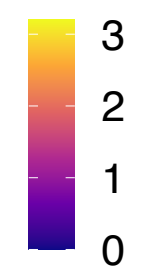**C**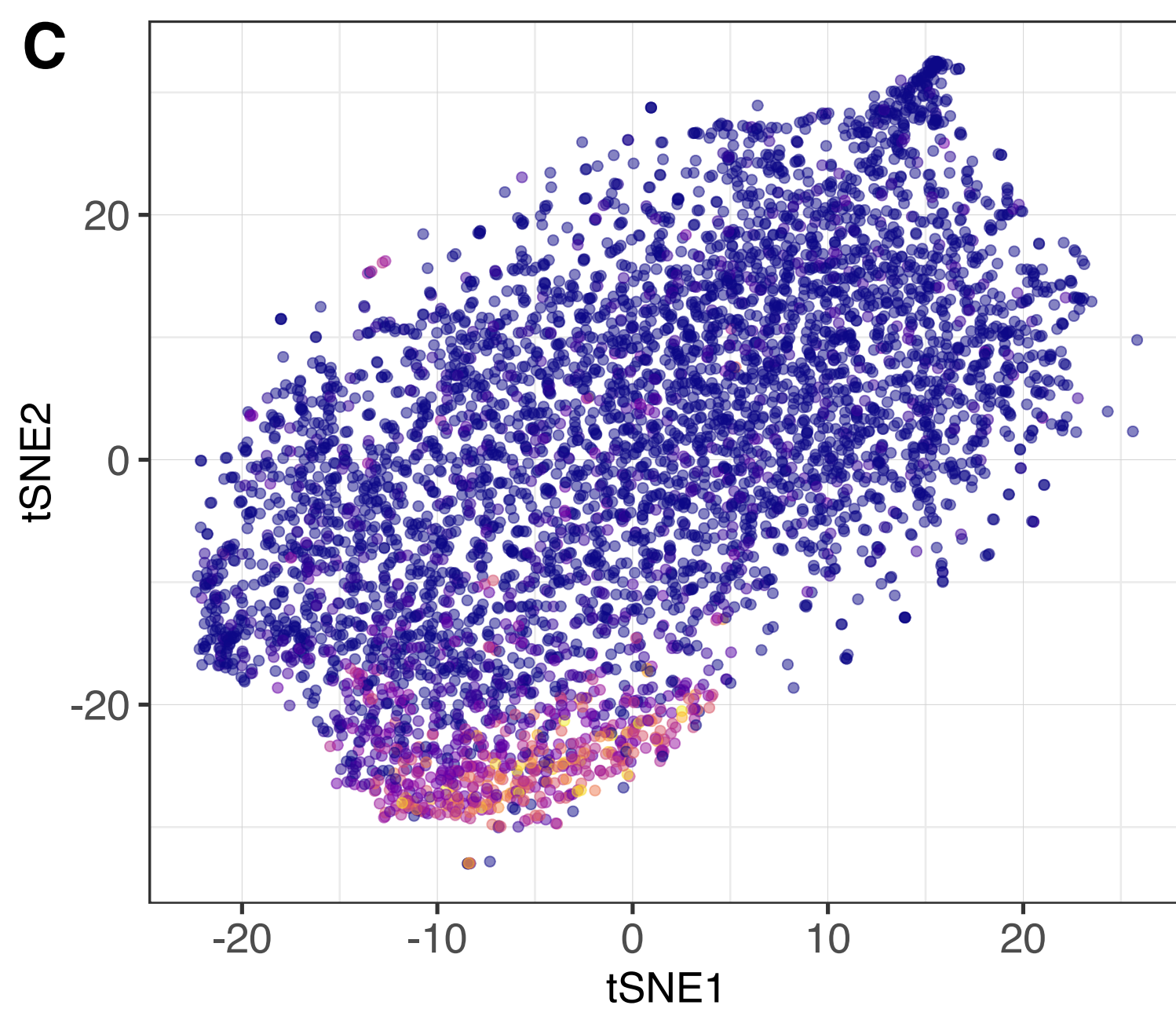

Proliferative signature (Travaglini et al.)

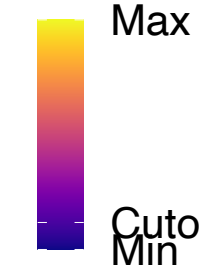

Supplement: Supplementary file 1 [file cells-11-01820-s001.zip › Supplement Figure S9.pdf]
